# Disentangling Climate Worry and Psychological Distress: Data From the UK Household Longitudinal Study

**DOI:** 10.1002/ijop.70221

**Published:** 2026-05-05

**Authors:** Vilja Helminen, Markus Jokela, Marko Elovainio

**Affiliations:** ^1^ Department of Psychology, Faculty of Medicine University of Helsinki Helsinki Finland

**Keywords:** climate beliefs, climate worry, mental health, political efficacy

## Abstract

Anxiety and worry about climate change have been associated with poorer mental health. We examined whether different climate beliefs, the cognitive component of climate anxiety, were associated with symptoms of psychological distress. We also tested whether political efficacy moderated the potential associations between climate beliefs and psychological distress, as perceptions of inadequate government response may amplify climate anxiety. We applied longitudinal data and cross‐lagged panel network models in the UK Household Longitudinal Study (*N* = 34,318). Our results showed that (1) the association between climate beliefs and psychological distress was bidirectional, (2) belief in responsibility was most clearly associated with increased distress while associations between belief in inevitability and the distress symptoms were more mixed, and (3) those who perceived that individuals can affect political decisions seemed to be most vulnerable to the negative effects of the climate crisis on mental health.

## 1 Introduction

Climate change related mental health has received increasing attention in the recent years. Most often the negative mental health implications of climate change have been approached from the perspective of climate anxiety, eco‐anxiety, or climate worry (e.g., Clayton 2021; Kurth and Pihkala 2022; Pihkala 2022). Many of such anxieties and worries are natural reactions to a large‐scale global environmental threat, not abnormal signs of psychopathology. Extreme perceptions of the climate threat might nonetheless be associated with negative mental health, as several studies have associated negative climate emotions with mental ill‐health (e.g., Boluda‐Verdú et al. 2022; Ramadan et al. 2023). However, many unanswered questions remain about the specific dynamics of climate related mental health: what is the direction of the association between climate perceptions and mental health, are there symptom‐specific associations, and do different climate related cognitions play a different role in the association?

Recent systematic reviews have shown climate worry and anxiety to be associated with increased symptoms of depression, anxiety, insomnia, and psychological distress (Boluda‐Verdú et al. 2022; Ramadan et al. 2023). However, it is unclear why these measures are linked to each other. As reliable longitudinal data is costly and difficult to collect most of the previous studies have been cross‐sectional. Longitudinal evidence is needed to assess whether psychological distress predisposes to climate worry, or vice versa. Experiences of domain general anxiety could predispose to assessing threats, including climate change, more negatively. The observed associations from cross‐sectional studies could also reflect underlying traits affecting both climate worry and mental health. For example, personality traits, especially neuroticism, are highly associated with mental health outcomes (e.g., Kang et al. 2023; Kotov et al. 2010) and could influence climate anxiety as well. As far as we know, only one study has examined whether prior mental health symptoms are associated with climate anxiety or worry. The study demonstrated that anxiety in adolescence was associated with greater climate worry in early adulthood (Vergunst et al. 2024), highlighting the need to account for prior mental health symptoms when examining the association of climate perceptions and mental health.

In addition to the lack of longitudinal data, most studies have not focused on the specifics of mental health symptoms (for reviews see Boluda‐Verdú et al. 2022; Ramadan et al. 2023). Most studies have examined sum scores of depression and anxiety (e.g., Schwartz et al. 2023; Searle and Gow 2010; Stanley et al. 2021). The traditional approach of using sum scores has several benefits, such as diminishing measurement error. On the other hand, complementary approaches that allow for examining the relationships between individual items, such as network models, may uncover important specifics in how mental health is associated with other constructs. The specific symptoms of mental health problems may be differently related to risk factors (Fried and Nesse 2015). A symptom‐specific approach could help to disentangle the heterogeneity of mental‐health symptoms related to climate worry.

Moreover, most previous studies have assessed how climate‐related feelings, or symptoms of climate anxiety and the impairment related to it, are associated with domain general mental health (e.g., Ogunbode et al. 2021; Schwartz et al. 2023; Searle and Gow 2010). Less is known about the role of different climate perceptions in the association between climate change and mental health. Examining how climate‐related cognitions such as negative beliefs and perceptions about the climate crisis may aid in building understanding of the dynamics between climate change and mental health. Cognitions—beliefs, thoughts, attitudes—are intertwined with emotions. Many widely accepted emotion theories include cognitions as either a component or antecedent of emotions. In general, these cognitive components are evaluative or factual beliefs about the object that elicits emotions (Moors and Scherer 2013; Reisenzein 2009). For example, social anxiety disorder is characterised by negative beliefs about social situations (Wong and Rapee 2016).

There is only little research mapping the specific beliefs that are central to climate related mental health. In qualitative studies, respondents have reported different kinds of consequences, irreversibility, collective inaction, and lack of control as some of the aspects of the climate crisis that make them anxious or worried (e.g., Ágoston et al. 2022; Schwartz et al. 2023; Verplanken et al. 2020). While one may be equally worried about the consequences of the climate crisis or the inevitability of it, these different forms of climate worry might have differing outcomes regarding, for example, mental health or climate action. For example, worry over the consequences of the crisis may motivate one to take action, while worry associated with the belief that the crisis cannot be prevented could benumb one instead. There are few scales of climate emotions that also address cognitions. The Inventory of Climate Emotions (Marczak et al. 2024) covers eight emotions tying them to perceptions of climate change: for example, the items assessing climate anger are presented as anger due to inadequate action from authorities and climate anxiety is tied to the severity of the threat climate change poses. The subscales of this scale have shown differential associations to mental health related outcomes in different countries. In a study using Norwegian and Irish samples most of the emotions were associated with more pronounced loneliness or alienation (Marczak et al. 2024). However, a recent study with a Chinese sample found that only climate guilt and climate alienation were associated with increase in mental health symptoms (Shao and Yu 2025). One previous study has investigated different forms of climate worry in relation to well‐being (Wullenkord and Ojala 2023). This study differentiated micro and macro worries, the former relating to climate crisis' negative effects on oneself and one's close social circle and the latter relating to the negative effects on the society and the planet at large. Both types of worries were associated with negative affect, although the association was stronger for micro worries than macro worries. However, these micro and macro worries were still quite similar in content, in that they referred to the consequences of the climate crisis. These studies offer tentative evidence that different perceptions of climate change may have differing implications for climate related mental health.

Climate related mental health may also be related to broader worldviews. Climate worry has been associated with feelings of being betrayed by the government and with perceptions of inadequate governmental response (Hickman et al. 2021). This is not surprising as the climate crisis is a societal problem that requires governmental action. Despite this, whether individuals' perceptions of their ability to influence societal decision‐making and the responsiveness of decision‐makers influence climate worry has not been studied extensively. Whether one feels they have power over political decision‐making in general could affect how they view and react to specific societal issues and crises. For example, individuals who feel their opinions are taken into account in politics may be more optimistic that climate change can still be mitigated and feel more optimistic about the future. The construct of political efficacy has been used for several decades to measure exactly these perceptions. Political efficacy consists of two dimensions: internal and external political efficacy (Craig et al. 1990). Internal political efficacy reflects how competent and able one thinks they are to take part in societal decision‐making. External political efficacy concerns the responsiveness of the decision‐making system: whether one thinks that citizens have real power to affect political decision‐making. Considering that climate change is a very topical political issue and the previous evidence that climate anxiety is related to perceptions of the governmental response (Hickman et al. 2021), reactions to the climate crisis might be partly modified by political efficacy.

In this study we examined (1) whether different climate beliefs are associated with psychological distress, (2) the temporality of these associations, and (3) if the associations differ between those who feel politically efficacious and those who do not. More specifically, we examined how climate crisis beliefs—beliefs about the inevitability of the crisis, personal responsibility, and the consequences of the crisis—are associated with later psychological distress, using longitudinal panel data from the United Kingdom. We used a network approach, which allowed us to inspect how climate crisis beliefs are related to specific symptoms rather than sum scores, and if there are differences in how specific symptoms relate to climate crisis beliefs. Furthermore, we examined how political efficacy might moderate these associations. We hypothesised that political efficacy could modify the association between climate worry and mental health, as climate change is a salient political issue and prior studies have connected climate anxiety to perceptions of the governmental response (Hickman et al. 2021). We expected that negative climate beliefs would be associated with increased psychological distress among individuals with low political efficacy, compared to those with high political efficacy.

## 2 Methods

### 2.1 Data

The data were from the UK Household Longitudinal Study (University of Essex, Institute for Social and Economic Research 2023), which is an ongoing study that covers around 40,000 households from the United Kingdom. The first data collection wave was in 2009, and there have since been 12 waves of data collection. We used data from waves 4 (collected in 2012–2014) and 10 (collected in 2018–2020) when the participants gave their responses to climate crisis perceptions, as well as political efficacy measured in Wave 3 (collected in 2011–2013). Respondents with missing data across all items in Wave 10 were excluded from the analyses. The resulting sample consisted of 34,318 respondents (44.82% men). The exact sample sizes are reported subsequently for each analysis and in the tables of edge weights of each network in the supplement. The mean, standard deviations, and missingness of the variables of interest, and the correlations between the variables are presented in the Supporting Information S1–S2.

### 2.2 Measurements

#### 2.2.1 Climate Crisis Beliefs

Climate crisis beliefs were measured using three items, ‘My actions contribute to climate change’ (personal responsibility), ‘Climate change is beyond control—it's too late to do anything’ (inevitability), and ‘People in the UK will be affected by climate change in the next 30 years’ (consequences). For personal responsibility and inevitability, the responses were on a scale 1–5, and were coded as 1 = completely disagree, 5 = completely agree. Responses for consequences were binary, coded as 0 = No, I do not believe this and 1 = Yes, I do believe this.

#### 2.2.2 Psychological Distress

Psychological distress was measured using the 12‐item General Health Questionnaire (GHQ‐12; Goldberg and Williams 1988). The GHQ‐12 consists of 12 items covering a range of symptoms, such as low mood and trouble sleeping. Each item asks the respondent about the frequency of each symptom in the past 2 weeks and is scored on a scale 0–3 where 3 indicates most distress. In our study, we use both the individual items, as well as a mean calculated across all 12 items resulting in a variable that ranged 0–3. The Cronbach's α for the scale in Wave 4 was 0.90 and in Wave 10 was 0.91.

#### 2.2.3 Political Efficacy

Internal and external political efficacy were measured (only in Wave 3) with two items each: ‘I consider myself to be well qualified to participate in politics’ and ‘I think I am better informed about politics than most people’ for internal political efficacy, and ‘Public officials don't care much about what people like me think’ and ‘People like me don't have any say in what the government does’ for external political efficacy. Each item was on a scale of 1 (‘Strongly agree’) to 5 (‘Strongly disagree’). These items closely resemble items included in the revised scales of internal and external efficacy outlined in Craig et al. (1990). The internal efficacy items were recoded so that higher scores indicated higher levels of efficacy. The correlation between two internal efficacy items was 0.63 and the two external efficacy items 0.59. We formed two categorical variables indicating high (median or above) or low (below median) internal and external political efficacy, by taking the mean of the two items and comparing it to the distribution across all respondents. These categorical variables were coded as 0 = below the median across all respondents, and 1 = at or above the median across all respondents.

#### 2.2.4 Covariates

Gender was measured as 0 (man) and 1 (woman) in both waves. Level of education was measured by recording the respondents' highest qualifications on a scale of Degree (6), Other higher degree (5), A‐level or equivalent (4), GCSE or equivalent (3), Other qualification (2), No qualification (1). For the purposes of the analysis comparing high and low education groups, high education was defined with those participants with degree or other higher degree.

### 2.3 Statistical Methods

First, we examined the cross‐sectional associations between climate crisis beliefs and mental health using linear regression analyses. We formed two models, one for Wave 4 (*n* = 22,867) and one for Wave 10 (*n* = 30,804), with all three climate crisis items as predictors of the GHQ‐12 mean. We used the GHQ‐12 mean to keep our approach comparable to most prior studies on climate anxiety and worry. Gender and level of education collected in the corresponding wave were included in the models as covariates.

Next, we examined the cross‐lagged associations between climate crisis beliefs and mental health using cross‐lagged panel network (CLPN) model (Wysocki et al. 2025). The CLPN model included all three climate crisis items and the individual GHQ‐12 items. In order to control for gender and level of education, we residualised the variables of interest for these background variables measured in Wave 10. Each variable at T2 (Wave 10) was regressed on itself and all other variables at T1 (Wave 4) using penalised maximum likelihood regression with a lasso penalty. This regularised regression estimation shrinks small regression paths to exactly zero and makes other paths larger resulting in a sparse network. We followed the procedure outlined by Wysocki et al. (2025), by fitting a series of univariate regression models, using 10‐fold cross‐validation to choose the tuning parameter *λ*. After this regularised regression step, we further pruned the model by re‐estimating the model in a structural equation modelling (SEM) framework using full‐information maximum likelihood estimation method. Only the statistically significant (*p* < 0.05) edges of the final model were interpreted. Although one of our climate crisis items was binary, it was treated as continuous in our analyses to obtain comparable estimates of the associations between the items. While this is not usually recommended, our approach can be justified as it might not bias the results too greatly (Hellevik 2009) while giving us the benefit of more readily interpretable coefficients. We conducted a sensitivity analysis without the binary item, the results of which are reported in the Supporting Information S7–S9. The exclusion of the binary item did not substantially affect the results. The fit of the model was good (*χ*2(df = 33) = 21.41, *p* = 0.940, RMSEA = 0.000 CFI = 1.00, TLI = 1.00). The regularised regression step required complete data, but the SEM estimation could handle missing observations. Therefore, the sample was 21,632 for the regularised regression step and 34,318 for the non‐regularised regression step.

In addition to visual examination of the networks, we calculated cross‐construct in‐predictability and out‐predictability for the climate crisis perceptions and psychological distress, again following the procedure outlined by Wysocki et al. (2025). In‐predictability is the proportion of variance in each variable at T2 that is accounted for by the other variables at T1. It reflects the extent to which a variable is predicted by other variables at a previous time point. Out‐predictability is the average across all the variables at T2 of the proportion of variance accounted for by each variable at T1. It reflects how well each variable at T1 predicts other variables at T2. Cross‐construct in‐predictability is then the extent to which a variable is predicted by all variables related to the other construct at a previous time point, whereas out‐predictability is the extent to which a variable predicts all variables related to the other construct at the later time point. Both in‐predictability and out‐predictability can range 0–1. We used bootstrapping to obtain estimates of the stability of these predictability metrics and report the mean and standard deviation of the metrics calculated from 500 bootstrapped samples.

To assess the moderating role of political efficacy on the association between climate crisis views and mental health, we formed groups of low and high internal political efficacy and groups of low and high external political efficacy (one standard deviation below/above the mean). We then estimated the CLPN model with the climate crisis perception items and all 12 GHQ‐12 items for each group and compared the low internal political efficacy group to the high internal political efficacy group, and the low external political efficacy group to the high external political efficacy group. The samples ranged 6804–13,317 in the regularised regression step and 8586–14,843 in the SEM estimation step.

To compare the networks between the political efficacy groups, we plotted the networks using an average layout between the compared groups and calculated Spearman correlations for the weighted adjacency matrices between the groups. This approach is descriptive but does not test whether the networks differ beyond sampling variability. We also examined cross‐construct predictability metrics of each node.

All analyses were run on R statistical software (v4.5.2; R Core Team, 2024) using RStudio. We used package *lm.beta* (Behrendt 2023) to obtain standardised regression coefficients in the cross‐sectional analyses, *glmnet* (Friedman et al. 2010) and *lavaan* (Rosseel 2012) to estimate the CLPN models, *bootnet* (Epskamp et al. 2018) to calculate the stability estimates of the predictability metrics, and *qgraph* (Epskamp et al. 2018) and *ggplot2* for visualisation. The analysis code is available at https://osf.io/vp4dh/.

## 3 Results

Climate crisis beliefs were cross‐sectionally associated with psychological distress within both waves. In Wave 4, all three beliefs were associated with distress: stronger belief in consequences (*B* = 0.03, *β* = 0.03, *p* < 0.001), personal responsibility (*B* = 0.01, *β* = 0.03, *p* < 0.001), and inevitability (*B* = 0.02, *β* = 0.05, *p* < 0.001) were all associated with higher distress. Consequences (*B* = 0.03, *β* = 0.02, *p* < 0.001) and inevitability (*B* = 0.03, *β* = 0.06, *p* < 0.001) were also associated with more distress in Wave 10, whereas the association between psychological distress and personal responsibility was not significant (*B* = 0.003, *β* = 0.006, *p* = 0.304).

**FIGURE 1:**
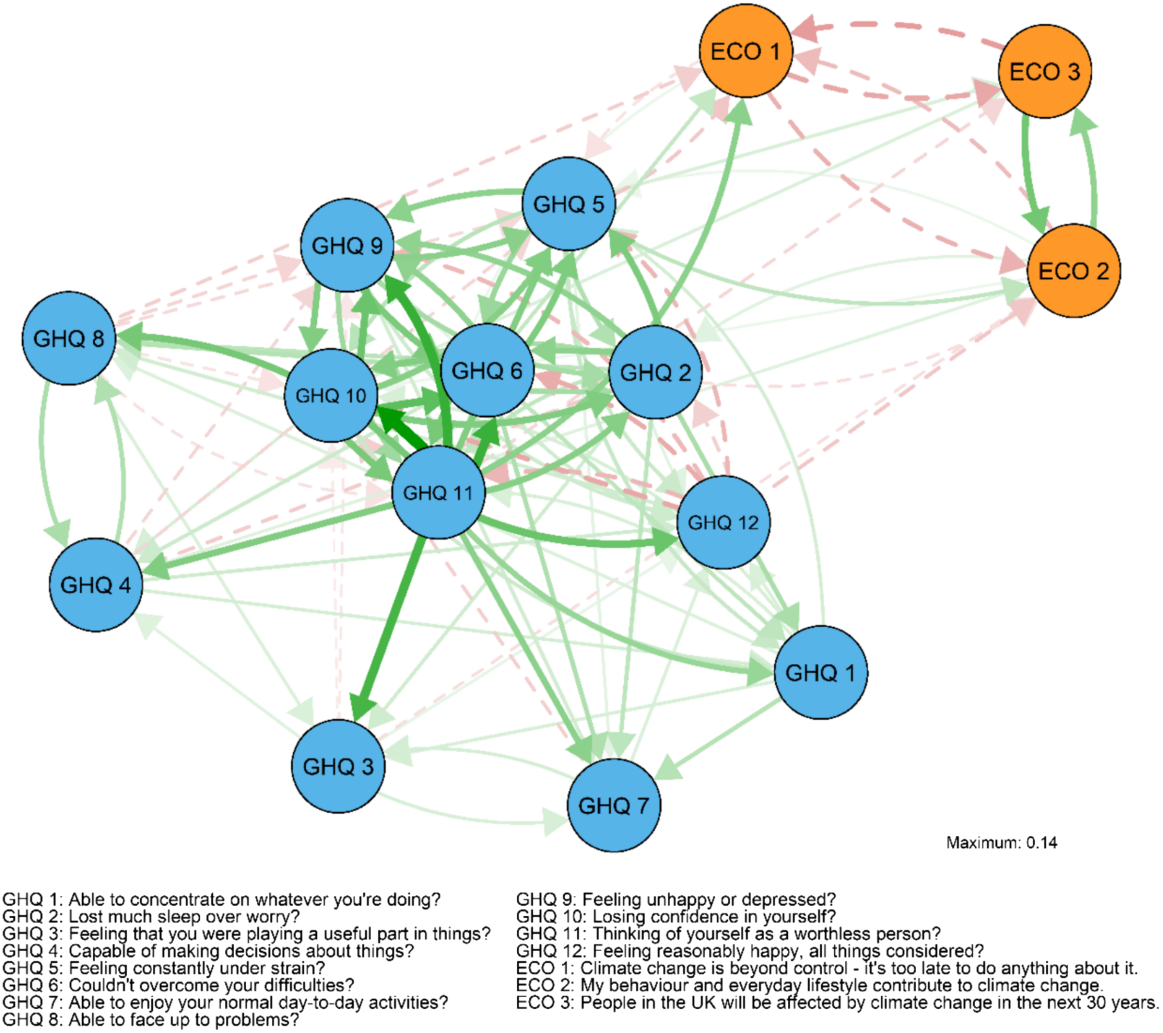
Cross‐lagged panel network, autoregressive edges removed. Green/solid lines represent positive and red/dashed lines negative edges. The directed arrows represent cross‐lagged associations where the node of origin is a variable measured in Wave 4 and the end node is a variable measured in Wave 10.

**FIGURE 2:**
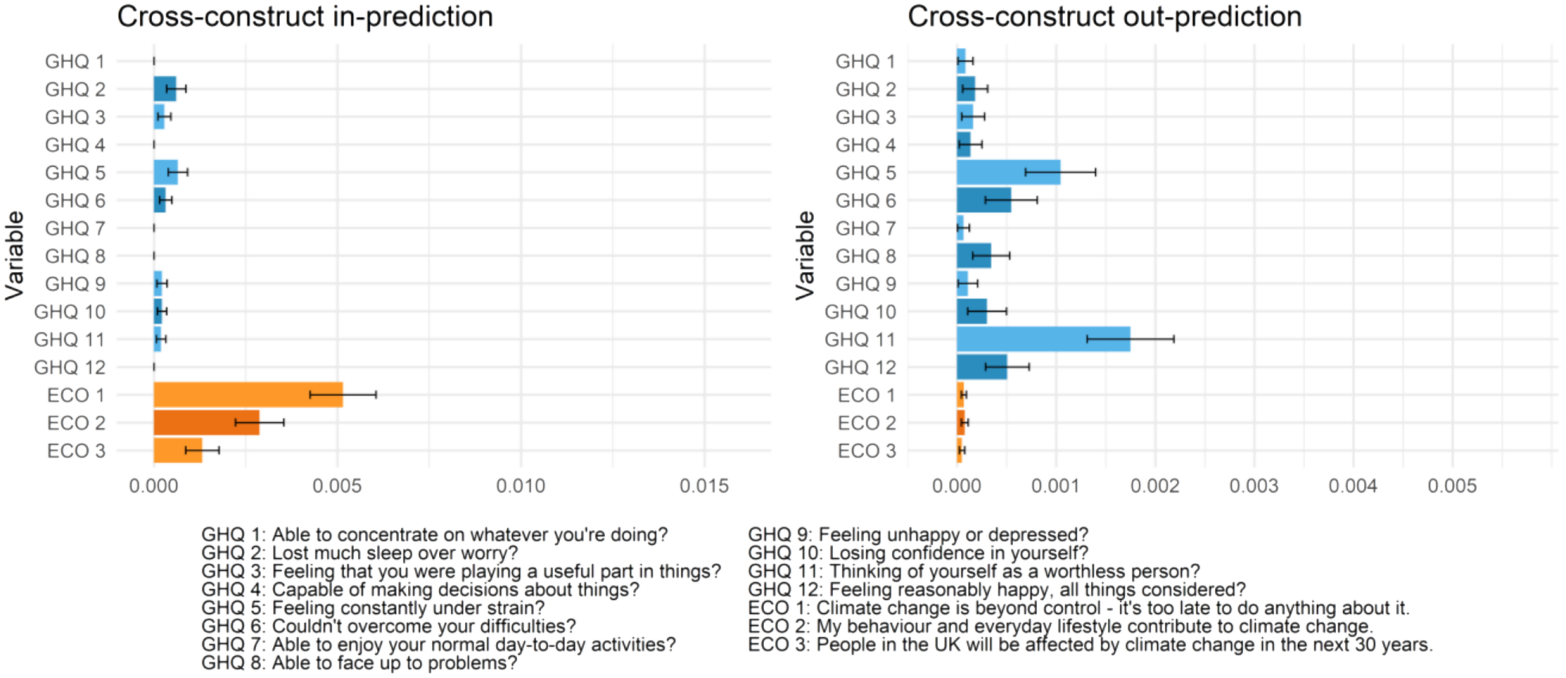
Cross‐construct predictability metrics for each node.

The CLPN model with all 12 individual psychological distress items is presented in Figure 1 and a table of the edge weights is presented in the Supporting Information S3. The associations between climate crisis beliefs and psychological distress items were relatively weak overall (absolute value of *β*_s_ = 0.01–0.07). All psychological distress symptoms were also relatively unreliable predictors of climate beliefs and vice versa. All items had either low out‐predictability indices or low stability in them (Figure 2). The magnitude of the edges indicates that distress may be more strongly associated with later beliefs than vice versa: the edges from distress to beliefs were on average stronger than the edges from beliefs to distress. The cross‐construct predictability metrics (Figure 2) also suggest that climate beliefs may be better predictors of psychological distress symptoms than vice versa. The average in‐prediction of each symptom remained low, with climate beliefs explaining less than 0.1% of the variance of each later distress symptom. The average in‐prediction of each climate belief was slightly higher with symptoms explaining 0.1%–0.5% of the variance of later beliefs.

The distress items that were best predicted by climate beliefs were losing sleep and feeling under strain. Belief in responsibility was associated with losing more sleep (*β* = 0.017) and feeling more under strain (*β* = 0.012). Belief in inevitability was associated with feeling *less* under strain (*β* = −0.016).

The cross‐construct in‐predictabilities suggest that previous psychological distress predicted belief in the inevitability and responsibility better than belief in the consequences of the crisis. Feeling under strain and feeling worthless were the best predictors of later climate crisis beliefs. Feeling under strain explained on average 0.1% of the variance in each belief item, and feeling worthless explained on average 0.2% of the variance in each belief item. Feeling under strain was associated with stronger belief in consequences (*β* = 0.022) and responsibility (*β* = 0.036) but weaker belief in inevitability (*β* = −0.032). Conversely, feeling worthless was associated with weaker belief in consequences (*β* = −0.023) and stronger belief in inevitability (*β* = 0.066). The association between feeling worthless and later belief in inevitability was the strongest among the cross‐construct associations.

As sensitivity analysis we also examined a model using only complete cases, the results of which are reported in the Supporting Information S4–S6. The results did not substantially change. The association between belief in responsibility and later feeling more under strain disappeared, and belief in inevitability had an additional association with later trouble overcoming difficulties. Feeling worthless also showed an additional association with later belief in responsibility.

We also examined how gender and level of education may modify the associations between climate beliefs and psychological distress by conducting the analyses separately for men and women (S10–S13) and those with and without higher education (S14–S17). Some gender differences emerged. Climate beliefs were more clearly associated with later distress among women, as indicated by a higher number of associations between the two and higher average cross‐construct out‐prediction indices of belief items. Belief in consequences was better predicted by prior distress among men compared to women, with distress symptoms explaining 0.4% of its variance for men and 0.2% for women. In both groups, the distress symptoms had both negative and positive associations with this belief. Most notable differences by level of education were that belief in responsibility was associated with later increased distress in the low education group, whereas it was not associated with any of the symptoms in the high education group. Beliefs in responsibility and consequences were better predicted by prior distress symptoms in the high education group.

**FIGURE 3:**
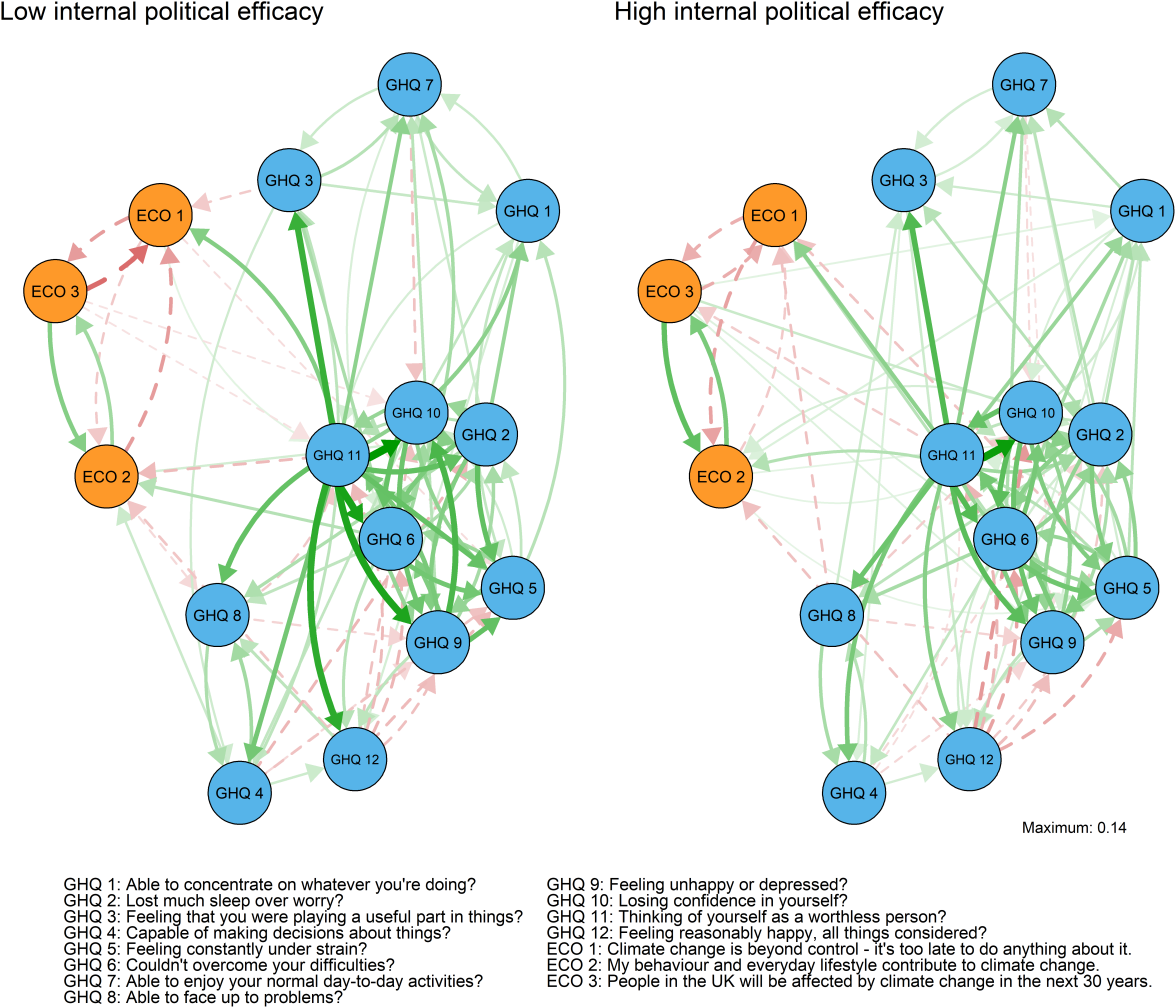
Low (left) versus high (right) internal political efficacy networks, autoregressive edges removed. Green/solid lines represent positive and red/dashed lines negative edges. The directed arrows represent cross‐lagged associations where the node of origin is a variable measured in Wave 4 and the end node is a variable measured in Wave 10.

The CLPN models for the low/high internal political efficacy groups are presented in Figure 3. A table of the edge weights and the cross‐construct predictability metrics in the Supporting Information S18–S20. The weighted adjacency matrices were highly correlated between the internal efficacy groups (*r* = 0.84 for all edges and *r* = 0.79 for edges excluding autoregressive edges). Overall, climate beliefs and distress items were relatively weak predictors of each other, as all items had low out‐predictability indices.

In the high efficacy group there climate beliefs were associated with increased distress, whereas in the low efficacy group the associations were mixed. In the high efficacy group all associations between beliefs and later distress symptoms were positive, for example, indicating more severe symptoms. Losing sleep was associated with prior belief in responsibility (*β* = 0.015) and consequences (*β* = 0.030). In addition, prior belief in responsibility was associated with feeling more under strain and losing confidence (*β*_s_ = 0.015). Prior belief in consequences was associated with overcoming difficulties (*β* = 0.019) and feeling unhappy (*β* = 0.018). Belief in inevitability was not associated with any of the symptoms. In the low efficacy group climate beliefs were associated with more and less severe symptoms, depending on the symptom. Belief in inevitability was associated with feeling less under strain (*β* = −0.019) and more worthless (*β* = 0.016). Belief in responsibility with increased loss of sleep (*β* = 0.025) and feeling more able to face up to problems (*β* = −0.021). Belief in consequences was associated with decreased distress: it had negative associations with losing confidence and feeling worthless (*β*_s_ = −0.017).

Feeling worthless was among the most reliable predictors of later climate beliefs in both groups, and in both groups its association with belief in inevitability (*β*_s_ = 0.063–0.068) was the strongest of all symptom‐belief associations. It was also associated with weaker belief in consequences in the high efficacy group (*β* = −0.028) and weaker belief in responsibility in the low efficacy group (*β* = −0.038). In the high efficacy group, feeling under strain also had relatively high out‐predictability, being associated with weaker belief in inevitability (*β* = −0.033) and stronger belief in responsibility (*β* = 0.042).

**FIGURE 4:**
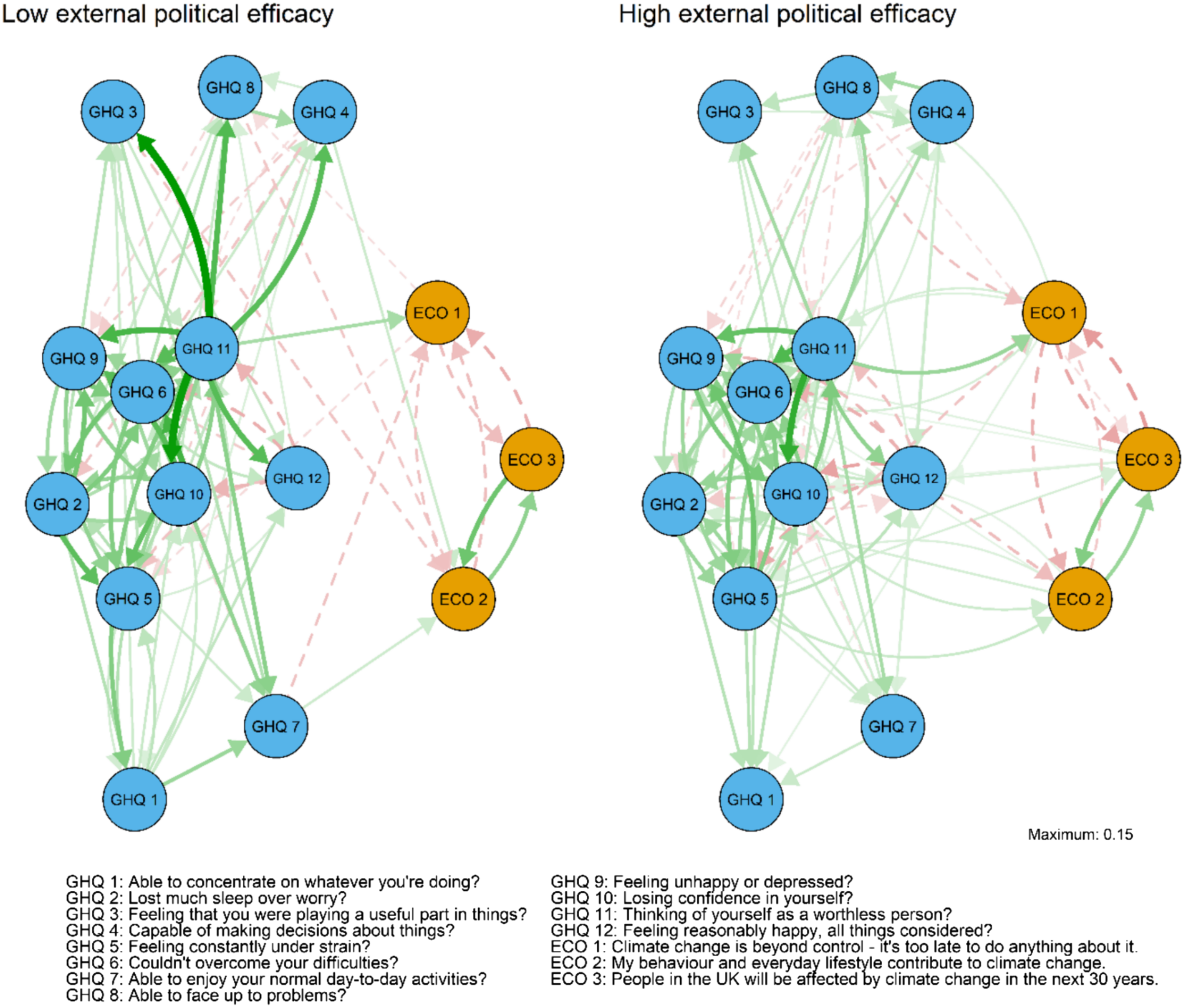
Low (left) versus high (right) external political efficacy networks, autoregressive edges removed. Green/solid lines represent positive and red/dashed lines negative edges. The directed arrows represent cross‐lagged associations where the node of origin is a variable measured in Wave 4 and the end node is a variable measured in Wave 10.

The CLPN models for the low/high external political efficacy groups are presented in Figure 4 and the accompanying cross‐construct predictability indices in Supporting Information S21–S23. The correlation for the weighted adjacency matrices between the external efficacy groups was lower than the internal efficacy groups (*r* = 0.78 for all edges and *r* = 0.71 for edges excluding autoregressive edges). As with the internal efficacy groups, climate beliefs and distress items were weak predictors of each other, with all items having low out‐predictability indices.

As with internal efficacy, prior beliefs were associated with increased distress in the high efficacy group. In the low efficacy group, belief in inevitability was associated with decreased distress. There were also fewer edges from previous climate beliefs to later psychological distress in the low efficacy group compared to the high efficacy group. In the high efficacy group, prior beliefs were associated with 9 of the 12 symptoms (*β*_s_ = 0.014–0.026). In the low efficacy group, only belief in inevitability was associated with later symptoms, more specifically feeling less under strain (*β* = −0.022) and more able to face up to problems (*β* = −0.020).

The contrast between the efficacy groups was not as stark concerning the associations between prior distress and later climate beliefs. Feeling worthless had the highest out‐predictability in both groups and was associated with stronger belief in inevitability (*β*_s_ = 0.053–0.061). In the high efficacy group, feeling under strain had also relatively high out‐prediction, being associated with weaker belief in inevitability (*β*_s_ = −0.036) and stronger beliefs in responsibility (*β* = 0.033) and consequences (*β* = 0.025).

## 4 Discussion

Our study offers longitudinal evidence on the associations between psychological distress and climate beliefs. Our results suggest that: (1) the dynamics between climate crisis beliefs and mental health are reciprocal, (2) these dynamics are complicated, and (3) political efficacy could moderate the association between climate worry and mental health.

First, our results indicate that while climate crisis beliefs are associated with later psychological distress, previous psychological distress is also associated with later climate beliefs. This is in line with a study which found anxiety in adolescence to be associated with climate worry in early adulthood (Vergunst et al. 2024). Our results point to prior distress possibly having a stronger association with climate beliefs than beliefs have with later distress. Although overall the associations between climate beliefs and distress symptoms remained relatively weak, the possibility that prior distress may influence climate beliefs more than vice versa calls for more longitudinal studies aiming to assess directionality of the associations between the mental health problems and climate worry or anxiety. Our results could also reflect some underlying dispositional trait affecting both, climate beliefs and psychological distress. This possibility should also be examined in future studies.

Second, our results point to rather complicated dynamics between climate beliefs and psychological distress comprising differing associations between specific beliefs and distress, and the associations between climate beliefs. The beliefs presented different patterns regarding the associations with later distress. Belief in responsibility was most often associated with increased distress across all our analyses, while belief in inevitability was associated either with decreased distress or both increased and decreased distress depending on the symptom. Belief in consequences was often not associated with later distress at all. Notably, the belief in consequences was measured on a dichotomous scale and we cannot rule out that the lack of associations with later distress is due to this. Our results point to the relative importance of belief in responsibility in the dynamics of climate‐related mental health. In line with our results, a prior study found that climate guilt, which shares similarities with our measure of belief in responsibility, was associated with later mental health symptoms (Shao and Yu 2025). However, our results differ from previous studies that have found emotions or worries related to the consequences of climate change to be associated with mental health‐related factors (Marczak et al. 2024; Wullenkord and Ojala 2023).

On the other hand, feeling worthless was especially strongly associated with later belief in inevitability as well as other symptoms of psychological distress later on: these associations were stronger than the associations between prior beliefs and later distress. Such strong associations between a central symptom of psychological distress and later beliefs further highlight the need to examine the possibility of reverse causation when examining climate related mental health.

Our results also point to climate beliefs being associated with each other. While belief in consequences and belief in responsibility were positively associated, belief in inevitability had negative associations with the other two beliefs. The negative link between responsibility and inevitability makes intuitive sense, as those who believe the climate crisis to be inevitable would also likely view that their actions are of little importance. Polls from the UK and the US imply that views highlighting the futility of changing one's behaviour and the wider inability or unwillingness of humankind to mitigate the crisis are not uncommon (Duffy 2021; Leiserowitz et al. 2018). However, the association between inevitability and consequences is less self‐evident. One tentative explanation may be the shared aspects of these climate fatalistic views with some common climate contrarian views. At least one study points to one of the most common forms of climate misinformation being the ineffectiveness or harmfulness of climate solutions (Coan et al. 2021).

The pattern of interactions between the climate beliefs was also reflected in how some of the symptoms were associated with climate beliefs in our overall model. Feeling under strain had reciprocal associations with both belief in inevitability and responsibility. Beliefs in responsibility may lead to feeling under strain, which in turn may strengthen these beliefs. This symptom was also reciprocally associated with *weaker* belief in inevitability. Feeling worthless, on the other hand, may exacerbate the aforementioned climate fatalistic views, as its association with stronger belief in inevitability was notably strong across all our analyses.

Third, our results suggest that perceptions of politics may play a role in climate‐related mental health. In both high efficacy groups, prior climate beliefs were clearly associated with increased distress, and in the low efficacy groups, the associations were either mixed (low internal efficacy) or negative (low external efficacy). This might indicate that climate beliefs have a negative association with mental health mainly among those who believe themselves capable of affecting societal matters and view the political system as responsive to the demands of the public, than among those who do not. The results regarding the role of political efficacy are surprising and unintuitive. As previous research has linked climate worry and anxiety to, for example, viewing governmental action as inadequate, it could be expected that those who feel unable and incapable of affecting decision‐making would be more at risk of negative mental health effects. One possibility is that our results reflect some other factors closely related to political efficacy. Those with low efficacy may, for example, feel more disenfranchised, which could then affect their views on climate change as a political issue. While previous studies on climate worry have not examined the role of political efficacy specifically, a study concluded that climate‐related self‐efficacy does not moderate the association between climate worries and mental health (Ogunbode et al. 2024). While climate‐related self‐efficacy and political efficacy share some aspects, political efficacy may capture some relevant political aspects of the climate‐related phenomena.

### 4.1 Limitations

The common limitations of generalisability apply to the results of our study: the sample came from the UK in 2012–2020 and it is possible that the specificities of the time and place influence our results. For example, extreme weather events could affect both views on climate change and mental health. There were severe winter storms and extreme flooding in the UK in 2013–2014 and 2019–2020 (Met Office, n.d.). The COVID pandemic may also have influenced participants' climate views and mental health in the 10th wave collected in 2018–2020.

Our study was limited to examining three climate crisis beliefs and cannot serve as direct evidence concerning climate beliefs more widely. Moreover, the measures of climate beliefs were not validated. Many prior studies on the topic use validated scales of climate anxiety, worry, or related constructs, limiting our ability to compare our results to prior studies.

The limitations of the data also affected our methodological choices. The CLPN lacks the ability to separate the within‐person effects from the between‐person effects, but the alternatives that do, such as random intercept cross‐lagged panel models (RI‐CLPM), would have required data from at least three data collection waves. The UK Household Longitudinal Study had included these climate crisis perceptions on only two waves, which made it impossible to use the recommended methods that do take stable traits into account. As such our results could reflect covarying traits rather than changes in climate beliefs being associated with changes in psychological distress. Considering that there are many traits (e.g., neuroticism) that can plausibly affect both mental health and perceptions of large‐scale societal threats, our results should be interpreted with caution. The UK Household Longitudinal Study collects data from all household members over the age of 16, which means that the individual respondents in our data are clustered to households. We were unable to account for household level clustering in our analyses, which may also bias our results. We compared the networks by calculating correlations between the edges: this approach is descriptive but does not test whether the networks differ beyond sampling variability.

Our data did not include a direct measure of the affects of the respondents. It is possible that agreement with the climate crisis items did not imply worry or anxiety over the matter for all respondents. This is especially true for the personal responsibility item which was phrased somewhat vaguely and might have been interpreted differently by different participants. Someone who does not believe that their actions contribute to climate change might do so because they have already taken radical actions in their everyday life to minimise the effects on climate change, or even because they simply do not believe the climate crisis to be true. Some studies suggest that negative climate emotions are more pronounced among the youth. Our data had a wide range of individuals of different ages, and it might be that the results would look rather different had we studied younger respondents individually. The lag between the time points may also impact our results. As there was some 6 years between the data collection waves, our study cannot account for how the dynamics between climate beliefs and psychological distress may appear on a shorter time‐scale. *Conclusions and recommendations for future studies*.

The most important conclusion based on our results is that climate anxiety or worry and mental health should be studied using longitudinal analyses and focusing on the mechanisms that underlie the previously observed associations. Overall, the associations between psychological distress and climate crisis beliefs were modest in our study, although there might be stronger associations on a smaller time scale that our study was not able to capture. Future studies should consider that cross‐sectional associations might be misleading and prioritise using longitudinal study designs.

Our results also suggest that the associations between mental health and climate crisis perceptions might not be straightforward, but rather climate worry can be associated with experiencing some symptoms less and others more. Similarly, different forms of climate worry might be differently associated with mental health. However, these results should be replicated in future studies. Future studies should also expand from this starting point and continue to examine different forms of climate worry with a more diverse set of climate beliefs and better measurement instruments.

Finally, as the climate crisis is not an individual level problem, climate crisis perceptions should also be assumed to be the result of the interplay between the individual and their society. Our study found some differences between political efficacy groups. Contrary to our expectations, those who feel that individuals have power to affect political matters might be most vulnerable to the negative effects of climate worry on mental health. We hope that future studies continue to take into account the societal aspect, by examining factors such as political efficacy or political trust in relation to climate worry in more depth.

## Author Contributions

**Vilja Helminen:** formal analysis, writing – original draft, writing – review and editing. **Markus Jokela:** methodology, writing – review and editing. **Marko Elovainio:** methodology, writing – review and editing.

## Funding

The authors were supported by Academy of Finland grants 329224, 339390, and 345186.

## Ethics Statement

The University of Essex Ethics Committee has approved all data collection on Understanding Society main study, COVID‐19 surveys and innovation panel waves, including asking consent for all data linkages except to health records. Requesting consent for health record linkage was approved at Wave 1 by the National Research Ethics Service (NRES) Oxfordshire REC A (08/H0604/124), at BHPS Wave 18 by the NRES Royal Free Hospital and Medical School (08/H0720/60) and at Wave 4 by NRES Southampton REC A (11/SC/0274). Approval for asking consent for health record linkage and for the collection of blood and subsequent serology testing in the March 2021 wave of the COVID‐19 study was obtained from London—City & East Research Ethics Committee (21/HRA/0644). Approval for the collection of biosocial data by trained nurses in Waves 2 and 3 of the main survey was obtained from the National Research Ethics Service (Understanding Society—UK Household Longitudinal Study: A Biosocial Component, Oxfordshire A REC, Reference: 10/H0604/2). The biosocial data collection at IP12 ‘Understanding Society Health Innovation Panel: Biomeasure and health data collection from the Innovation Panel of the UK Household Longitudinal Study’ was approved by East of England—Essex Research Ethics Committee, Ref 19/EE/0146.

## Conflicts of Interest

The authors declare no conflicts of interest.

## Supporting information


**Data S1:** Means, standard deviations, and missingness of the variables of interest.
**S2** Correlations between variables of interest.
**S3** The edge weights of the cross‐lagged panel network of the whole data.
**S4** The cross‐lagged panel network model using complete cases.
**S5** The edge weights of the cross‐lagged panel network with only complete cases.
**S6** Cross‐construct predictability (in) and influence (out) for each node using only complete cases.
**S7** The cross‐lagged panel network model excluding the binary item.
**S8** The edge weights of the cross‐lagged panel network excluding the binary item.
**S9** Cross‐construct predictability (in) and influence (out) for each node of the network model excluding the binary item.
**S10** The cross‐lagged panel network models for men and women.
**S11** The edge weights of the cross‐lagged panel network for men.
**S12** The edge weights of the cross‐lagged panel network excluding the binary item for women.
**S13** Cross‐construct predictability (in) and influence (out) for each node for men versus women.
**S14** The cross‐lagged panel network models for high and low education groups.
**S15** The edge weights of the cross‐lagged panel network for the high education group.
**S16** The edge weights of the cross‐lagged panel network for the low education group.
**S17** Cross‐construct predictability (in) and influence (out) for each node in low versus high education groups.
**S18** The edge weights of the cross‐lagged panel network for the high internal political efficacy group.
**S19** The edge weights of the cross‐lagged panel network for the low internal political efficacy group.
**S20** Cross‐construct predictability metrics for each node in low versus high internal political efficacy groups.
**S21** The edge weights of the cross‐lagged panel network for the high external political efficacy group.
**S22** The edge weights of the cross‐lagged panel network for the low external political efficacy group.
**S23** Cross‐construct predictability (in) and influence (out) for each node in low versus high external political efficacy groups.

## Data Availability

The data that support the findings of this study are openly available from the UK Data Service at https://ukdataservice.ac.uk/, reference number 10.5255/UKDA‐SN‐6614‐17.

## References

[ijop70221-bib-0001] Ágoston, C. , B. Csaba , B. Nagy , et al. 2022. “Identifying Types of Eco‐Anxiety, Eco‐Guilt, Eco‐Grief, and Eco‐Coping in a Climate‐Sensitive Population: A Qualitative Study.” International Journal of Environmental Research and Public Health 19, no. 4: 2461. 10.3390/ijerph19042461. 35206648 PMC8875433

[ijop70221-bib-0002] Behrendt, S. 2023. “lm.beta: Add Standardized Regression Coefficients to Linear‐Model‐Objects. R Package Version 1.7‐2.” https://CRAN.R‐project.org/package=lm.beta.

[ijop70221-bib-0003] Boluda‐Verdú, I. , M. Senent‐Valero , M. Casas‐Escolano , A. Matijasevich , and M. Pastor‐Valero . 2022. “Fear for the Future: Eco‐Anxiety and Health Implications, a Systematic Review.” Journal of Environmental Psychology 84: 101904. 10.1016/j.jenvp.2022.101904.

[ijop70221-bib-0004] Clayton, S. 2021. “Climate Change and Mental Health.” Current Environmental Health Reports 8, no. 1: 1–6. 10.1007/s40572-020-00303-3. 33389625

[ijop70221-bib-0038] Coan, T. G. , C. Boussalis , J. Cook , and M. O. Nanko . 2021. “Computer‐Assisted Classification of Contrarian Claims About Climate Change.” Scientific Reports 11, no. 1. 10.1038/s41598-021-01714-4. PMC8595491 34785707

[ijop70221-bib-0005] Craig, S. C. , R. G. Niemi , and G. E. Silver . 1990. “Political Efficacy and Trust: A Report on the NES Pilot Study Items.” Political Behavior 12, no. 3: 289–314. 10.1007/BF00992337.

[ijop70221-bib-0034] Duffy, B. 2021. “Who cares about climate change? Attitudes across the generations.” https://www.kcl.ac.uk/policy‐institute/assets/who‐cares‐about‐climate‐change.pdf.

[ijop70221-bib-0006] Epskamp, S. , D. Borsboom , and E. I. Fried . 2018. “Estimating Psychological Networks and Their Accuracy: A Tutorial Paper.” Behavior Research Methods 50: 195–212. 28342071 10.3758/s13428-017-0862-1 PMC5809547

[ijop70221-bib-0007] Fried, E. I. , and R. M. Nesse . 2015. “Depression Sum‐Scores Don't Add Up: Why Analyzing Specific Depression Symptoms Is Essential.” BMC Medicine 13, no. 1: 72. 10.1186/s12916-015-0325-4. 25879936 PMC4386095

[ijop70221-bib-0008] Friedman, J. , T. Hastie , and R. Tibshirani . 2010. “Regularization Paths for Generalized Linear Models via Coordinate Descent.” Journal of Statistical Software 33, no. 1: 1–22. 10.18637/jss.v033.i01. 20808728 PMC2929880

[ijop70221-bib-0009] Goldberg, D. P. , and P. Williams . 1988. A User's Guide to the General Health Questionnaire. NFER‐NELSON.

[ijop70221-bib-0010] Hellevik, O. 2009. “Linear Versus Logistic Regression When the Dependent Variable Is a Dichotomy.” Quality & Quantity 43, no. 1: 59–74. 10.1007/s11135-007-9077-3.

[ijop70221-bib-0011] Hickman, C. , E. Marks , P. Pihkala , et al. 2021. “Climate Anxiety in Children and Young People and Their Beliefs About Government Responses to Climate Change: A Global Survey.” Lancet Planetary Health 5, no. 12: e863–e873. 10.1016/S2542-5196(21)00278-3. 34895496

[ijop70221-bib-0036] Kang, W. , F. Steffens , S. Pineda , K. Widuch , and A. Malvaso . 2023. “Personality Traits and Dimensions of Mental Health.” Scientific Reports 13, no. 1. 10.1038/s41598-023-33996-1. PMC10151354 37127723

[ijop70221-bib-0035] Kotov, R. , W. Gamez , F. Schmidt , and D. Watson . 2010. “Linking “big” Personality Traits to Anxiety, Depressive, and Substance use Disorders: A Meta‐Analysis.” Psychological Bulletin 136, no. 5: 768–821. 10.1037/a0020327. 20804236

[ijop70221-bib-0012] Kurth, C. , and P. Pihkala . 2022. “Eco‐Anxiety: What It Is and Why It Matters.” Frontiers in Psychology 13: 981814. 10.3389/fpsyg.2022.981814. 36211934 PMC9537110

[ijop70221-bib-0037] Leiserowitz, A. , E. Maibach , C. Roser‐Renouf , S. Rosenthal , M. Cutler , and J. Kotcher . 2018. Climate change in the American mind: March 2018. Yale University and George Mason University. New Haven, CT: Yale Program on Climate Change Communication.

[ijop70221-bib-0013] Marczak, M. , M. Wierzba , B. Kossowski , A. Marchewka , R. Morote , and C. A. Klöckner . 2024. “Emotional Responses to Climate Change in Norway and Ireland: A Validation of the Inventory of Climate Emotions (ICE) in Two European Countries and an Inspection of Its Nomological Span.” Frontiers in Psychology 15: 1211272. 10.3389/fpsyg.2024.1211272. 38390416 PMC10881694

[ijop70221-bib-0014] Met Office . n.d. “Past Weather Events. Past Weather Events.” https://weather.metoffice.gov.uk/learn‐about/past‐uk‐weather‐events.

[ijop70221-bib-0015] Moors, A. , and K. R. Scherer . 2013. “The Role of Appraisal in Emotion.” In Handbook of Cognition and Emotion, edited by M. D. Robinson , E. R. Watkins , and E. Harmon‐Jones , 135–155. Guilford Press.

[ijop70221-bib-0016] Ogunbode, C. A. , S. Pallesen , G. Böhm , et al. 2021. “Negative Emotions About Climate Change Are Related to Insomnia Symptoms and Mental Health: Cross‐Sectional Evidence From 25 Countries.” Current Psychology 42: 845–854. 10.1007/s12144-021-01385-4.

[ijop70221-bib-0033] Ogunbode, C. A. , K. Salmela‐Aro , D. A. Maran , et al. 2024. “Do Neuroticism and Efficacy Beliefs Moderate the Relationship Between Climate Change Worry and Mental Wellbeing?” Journal of Affective Disorders 364: 37–40. 10.1016/j.jad.2024.08.018. 39134152

[ijop70221-bib-0017] Pihkala, P. 2022. “Toward a Taxonomy of Climate Emotions.” Frontiers in Climate 3: 738154. 10.3389/fclim.2021.738154.

[ijop70221-bib-0039] Ramadan, R. , A. Randell , S. Lavoie , et al. 2023. “Empirical Evidence for Climate Concerns, Negative Emotions and Climate‐Related Mental Ill‐Health in Young People: A Scoping Review.” Early Intervention in Psychiatry 17, no. 6: 537–563. Portico. 10.1111/eip.13374. 36641809

[ijop70221-bib-0019] Reisenzein, R. 2009. “Emotions as Metarepresentational States of Mind: Naturalizing the Belief–Desire Theory of Emotion.” Cognitive Systems Research 10, no. 1: 6–20. 10.1016/j.cogsys.2008.03.001.

[ijop70221-bib-0020] Rosseel, Y. 2012. “Lavaan: An R Package for Structural Equation Modeling.” Journal of Statistical Software 48, no. 2: 1–36. 10.18637/jss.v048.i02.

[ijop70221-bib-0021] Schwartz, S. E. O. , L. Benoit , S. Clayton , M. F. Parnes , L. Swenson , and S. R. Lowe . 2023. “Climate Change Anxiety and Mental Health: Environmental Activism as Buffer.” Current Psychology 42, no. 20: 16708–16721. 10.1007/s12144-022-02735-6. PMC8883014 35250241

[ijop70221-bib-0023] Searle, K. , and K. Gow . 2010. “Do Concerns About Climate Change Lead to Distress?” International Journal of Climate Change Strategies and Management 2, no. 4: 362–379. 10.1108/17568691011089891.

[ijop70221-bib-0024] Shao, L. , and G. Yu . 2025. “Psychometric Properties of the Inventory of Climate Emotions and Its Links With Mental Health and Climate Actions in a Chinese Sample.” Journal of Environmental Psychology 107: 102751. 10.1016/j.jenvp.2025.102751.

[ijop70221-bib-0025] Stanley, S. K. , T. L. Hogg , Z. Leviston , and I. Walker . 2021. “From Anger to Action: Differential Impacts of Eco‐Anxiety, Eco‐Depression, and Eco‐Anger on Climate Action and Wellbeing.” Journal of Climate Change and Health 1: 100003. 10.1016/j.joclim.2021.100003.

[ijop70221-bib-0026] University of Essex, Institute for Social and Economic Research . 2023. Understanding Society: Waves 1–13, 2009–2022 and Harmonised BHPS: Waves 1–18, 1991–2009. [Data Collection]. SN: 6614. 18th ed. UK Data Service. 10.5255/UKDA-SN-6614-19.

[ijop70221-bib-0027] Vergunst, F. , C. M. Prentice , M. Orri , et al. 2024. “Association of Youth Climate Change Worry With Present and Past Mental Health Symptoms: A Longitudinal Population‐Based Study.” Climatic Change 177, no. 10: 153. 10.1007/s10584-024-03807-1.

[ijop70221-bib-0028] Verplanken, B. , E. Marks , and A. I. Dobromir . 2020. “On the Nature of Eco‐Anxiety: How Constructive or Unconstructive Is Habitual Worry About Global Warming?” Journal of Environmental Psychology 72: 101528. 10.1016/j.jenvp.2020.101528.

[ijop70221-bib-0030] Wong, Q. J. J. , and R. M. Rapee . 2016. “The Aetiology and Maintenance of Social Anxiety Disorder: A Synthesis of Complementary Theoretical Models and Formulation of a New Integrated Model.” Journal of Affective Disorders 203: 84–100. 10.1016/j.jad.2016.05.069. 27280967

[ijop70221-bib-0031] Wullenkord, M. C. , and M. Ojala . 2023. “Climate‐Change Worry Among Two Cohorts of Late Adolescents: Exploring Macro and Micro Worries, Coping, and Relations to Climate Engagement, Pessimism, and Well‐Being.” Journal of Environmental Psychology 90: 102093. 10.1016/j.jenvp.2023.102093.

[ijop70221-bib-0032] Wysocki, A. , I. McCarthy , R. Van Bork , A. O. J. Cramer , and M. Rhemtulla . 2025. “Cross‐Lagged Panel Networks.” Advances in Psychology 2, no. 1: e739621. 10.56296/aip00037.

