# Supplementary material for: Disentangling Climate Worry and Psychological Distress: Data From the UK Household Longitudinal Study
Source: Int J Psychol. 2026 May 5;61:e70221. doi: 10.1002/ijop.70221 (PMC13143408; doi:10.1002/ijop.70221)
Supplement: Supplementary file 1 — Data S1: Means, standard deviations, and missingness of the variables of interest. S2 Correlations between variables of interest. S3 The edge weights of the cross‐lagged panel network of the whole data. S4 The cross‐lagged panel network model using complete cases. S5 The edge weights of the cross‐lagged panel network with only complete cases. S6 Cross‐construct predictability (in) and influence (out) for each node using only complete cases. S7 The cross‐lagged panel network model excluding the binary item. S8 The edge weights of the cross‐lagged panel network excluding the binary item. S9 Cross‐construct predictability (in) and influence (out) for each node of the network model excluding the binary item. S10 The cross‐lagged panel network models for men and women. S11 The edge weights of the cross‐lagged panel network for men. S12 The edge weights of the cross‐lagged panel network excluding the binary item for women. S13 Cross‐construct predictability (in) and influence (out) for each node for men versus women. S14 The cross‐lagged panel network models for high and low education groups. S15 The edge weights of the cross‐lagged panel network for the high education group. S16 The edge weights of the cross‐lagged panel network for the low education group. S17 Cross‐construct predictability (in) and influence (out) for each node in low versus high education groups. S18 The edge weights of the cross‐lagged panel network for the high internal political efficacy group. S19 The edge weights of the cross‐lagged panel network for the low internal political efficacy group. S20 Cross‐construct predictability metrics for each node in low versus high internal political efficacy groups. S21 The edge weights of the cross‐lagged panel network for the high external political efficacy group. S22 The edge weights of the cross‐lagged panel network for the low external political efficacy group. S23 Cross‐construct predictability (in) and influence (out) for each node in low versus high e [file IJOP-61-e70221-s001.docx]

**Supplementary Information**

**S1** Means, standard deviations, and missingness of the variables of interest

|  | | | | | | | | | | | | |
| --- | --- | --- | --- | --- | --- | --- | --- | --- | --- | --- | --- | --- |
| Variable | Wave 3 | | | | Wave 4 | | | | Wave 10 | | | |
|  | Mean | sd | Missing | Missing % | Mean | sd | Missing | Missing % | Mean | sd | Missing | Missing % |
| IPE | 2.73 | 0.94 | 10056 | 29 |  |  |  |  |  |  |  |  |
| EPE | 2.66 | 0.9 | 10120 | 29 |  |  |  |  |  |  |  |  |
| GHQ mean |  |  |  |  | 0.91 | 0.45 | 11054 | 32 | 0.95 | 0.47 | 2420 | 7 |
| GHQ 1 |  |  |  |  | 2.15 | 0.51 | 11025 | 32 | 2.16 | 0.51 | 2182 | 6 |
| GHQ 2 |  |  |  |  | 1.78 | 0.78 | 11023 | 32 | 1.83 | 0.76 | 2160 | 6 |
| GHQ 3 |  |  |  |  | 2.07 | 0.55 | 11038 | 32 | 2.11 | 0.55 | 2209 | 6 |
| GHQ 4 |  |  |  |  | 2.02 | 0.44 | 11028 | 32 | 2.05 | 0.45 | 2177 | 6 |
| GHQ 5 |  |  |  |  | 1.97 | 0.79 | 11027 | 32 | 1.99 | 0.78 | 2179 | 6 |
| GHQ 6 |  |  |  |  | 1.75 | 0.75 | 11028 | 32 | 1.79 | 0.75 | 2182 | 6 |
| GHQ 7 |  |  |  |  | 2.12 | 0.53 | 11025 | 32 | 2.16 | 0.53 | 2159 | 6 |
| GHQ 8 |  |  |  |  | 2.05 | 0.46 | 11030 | 32 | 2.08 | 0.47 | 2172 | 6 |
| GHQ 9 |  |  |  |  | 1.81 | 0.82 | 11028 | 32 | 1.88 | 0.81 | 2171 | 6 |
| GHQ 10 |  |  |  |  | 1.71 | 0.8 | 11028 | 32 | 1.77 | 0.81 | 2161 | 6 |
| GHQ 11 |  |  |  |  | 1.41 | 0.7 | 11031 | 32 | 1.48 | 0.73 | 2170 | 6 |
| GHQ 12 |  |  |  |  | 2.05 | 0.56 | 11027 | 32 | 2.09 | 0.56 | 2181 | 6 |
| ECO 1 |  |  |  |  | 2.62 | 0.96 | 11134 | 32 | 2.49 | 0.95 | 2353 | 7 |
| ECO 2 |  |  |  |  | 3.35 | 0.91 | 11114 | 32 | 3.39 | 0.91 | 2348 | 7 |
| ECO 3 |  |  |  |  | 0.79 | 0.4 | 11300 | 33 | 0 | 0.36 | 3154 | 9 |

**S2** Correlations between variables of interest

**
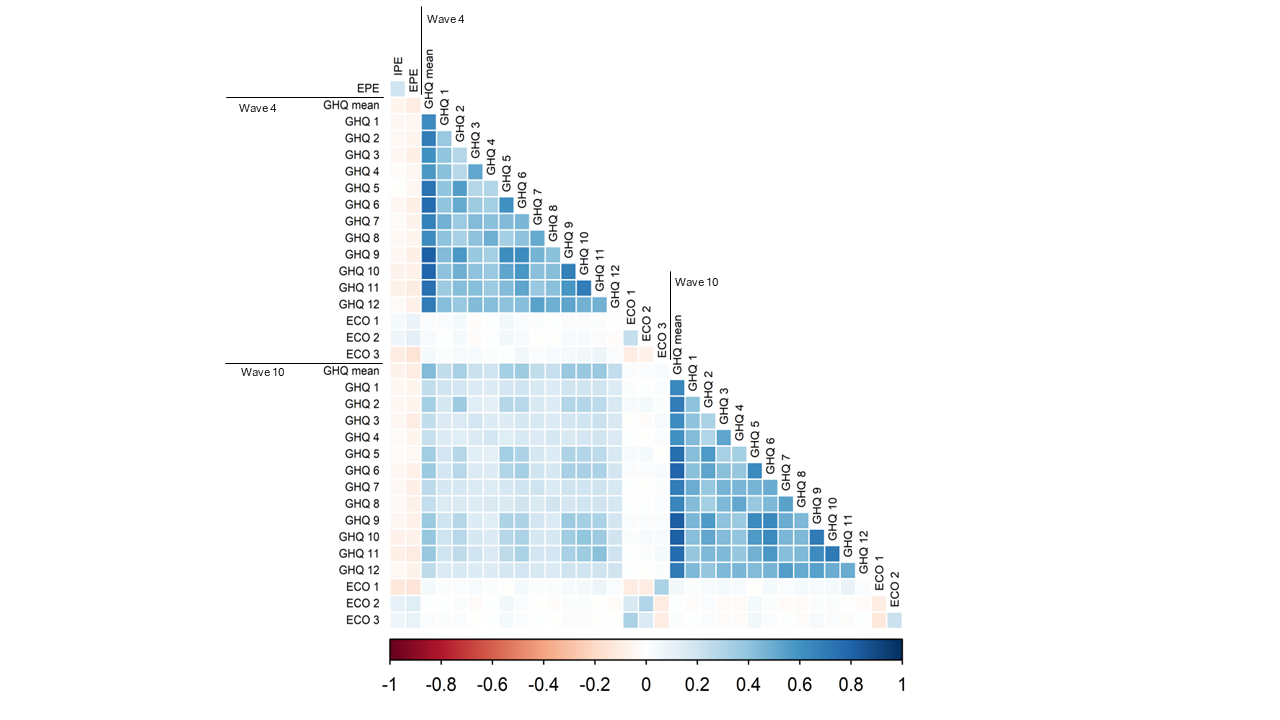
**

**S3** The edge weights of the cross-lagged panel network of the whole data

|  | Wave 10 | | | | | | | | | | | | | | |
| --- | --- | --- | --- | --- | --- | --- | --- | --- | --- | --- | --- | --- | --- | --- | --- |
| Wave 4 | GHQ 1 | GHQ 2 | GHQ 3 | GHQ 4 | GHQ 5 | GHQ 6 | GHQ 7 | GHQ 8 | GHQ 9 | GHQ 10 | GHQ 11 | GHQ 12 | ECO 1 | ECO 2 | ECO 3 |
| GHQ1 | 0.088 | 0.000 | 0.023 | 0.000 | 0.025 | 0.000 | 0.041 | 0.020 | 0.017 | 0.023 | 0.022 | 0.022 | 0.000 | 0.000 | 0.000 |
| GHQ 2 | 0.047 | 0.228 | 0.027 | 0.000 | 0.074 | 0.064 | 0.037 | 0.038 | 0.063 | 0.049 | 0.035 | 0.018 | 0.000 | -0.019 | 0.000 |
| GHQ 3 | 0.000 | 0.000 | 0.076 | 0.019 | 0.000 | 0.000 | 0.022 | 0.000 | -0.014 | -0.012 | 0.000 | 0.014 | 0.000 | -0.018 | 0.000 |
| GHQ 4 | 0.025 | -0.029 | 0.000 | 0.090 | -0.024 | 0.000 | 0.000 | 0.045 | -0.024 | 0.000 | 0.000 | 0.028 | 0.000 | 0.019 | 0.000 |
| GHQ 5 | 0.032 | 0.044 | 0.000 | 0.000 | 0.168 | 0.057 | 0.016 | 0.000 | 0.065 | 0.040 | 0.000 | 0.030 | -0.032 | 0.036 | 0.022 |
| GHQ 6 | 0.037 | 0.051 | 0.031 | 0.029 | 0.073 | 0.135 | 0.045 | 0.042 | 0.063 | 0.070 | 0.077 | 0.027 | 0.033 | 0.000 | 0.000 |
| GHQ 7 | 0.000 | 0.000 | 0.024 | 0.000 | 0.000 | 0.000 | 0.078 | 0.000 | 0.000 | -0.028 | 0.000 | 0.021 | 0.000 | 0.000 | 0.000 |
| GHQ 8 | 0.000 | 0.000 | 0.022 | 0.048 | -0.022 | 0.000 | 0.000 | 0.080 | -0.024 | -0.017 | -0.017 | 0.000 | -0.028 | 0.000 | 0.000 |
| GHQ 9 | 0.000 | 0.051 | 0.000 | 0.000 | 0.066 | 0.074 | 0.026 | 0.000 | 0.166 | 0.071 | 0.054 | 0.028 | 0.000 | 0.000 | 0.000 |
| GHQ 10 | 0.030 | 0.072 | 0.000 | 0.023 | 0.068 | 0.092 | 0.000 | 0.000 | 0.083 | 0.195 | 0.076 | 0.000 | 0.000 | 0.000 | 0.021 |
| GHQ 11 | 0.058 | 0.068 | 0.104 | 0.071 | 0.071 | 0.112 | 0.066 | 0.077 | 0.113 | 0.144 | 0.301 | 0.084 | 0.066 | 0.000 | -0.023 |
| GHQ 12 | 0.000 | -0.026 | 0.000 | 0.000 | -0.041 | -0.047 | 0.000 | 0.026 | -0.040 | -0.049 | -0.041 | 0.068 | 0.000 | -0.032 | 0.000 |
| ECO 1 | 0.000 | 0.000 | 0.000 | 0.000 | -0.016 | 0.000 | 0.000 | 0.000 | 0.000 | 0.000 | 0.011 | 0.000 | 0.289 | -0.044 | -0.049 |
| ECO 2 | 0.000 | 0.017 | 0.000 | 0.000 | 0.012 | 0.000 | 0.000 | 0.000 | 0.000 | 0.011 | 0.000 | 0.000 | -0.038 | 0.257 | 0.063 |
| ECO 3 | 0.000 | 0.000 | 0.000 | 0.000 | 0.000 | 0.000 | 0.000 | 0.000 | 0.000 | 0.000 | 0.000 | 0.000 | -0.056 | 0.079 | 0.290 |

Note. The estimates are edge weights of variables measured in wave 4 predicting variables measured in wave 10. Cross-construct associations highlighted. Model fit: χ2(df = 33) = 21.41, p = .940, RMSEA = .000 CFI = 1.00, TLI = 1.00. n = 21,632 in the regularized regression step and n = 34,318 in the non-regularized regression step.

**S4** The cross-lagged panel network model using complete cases


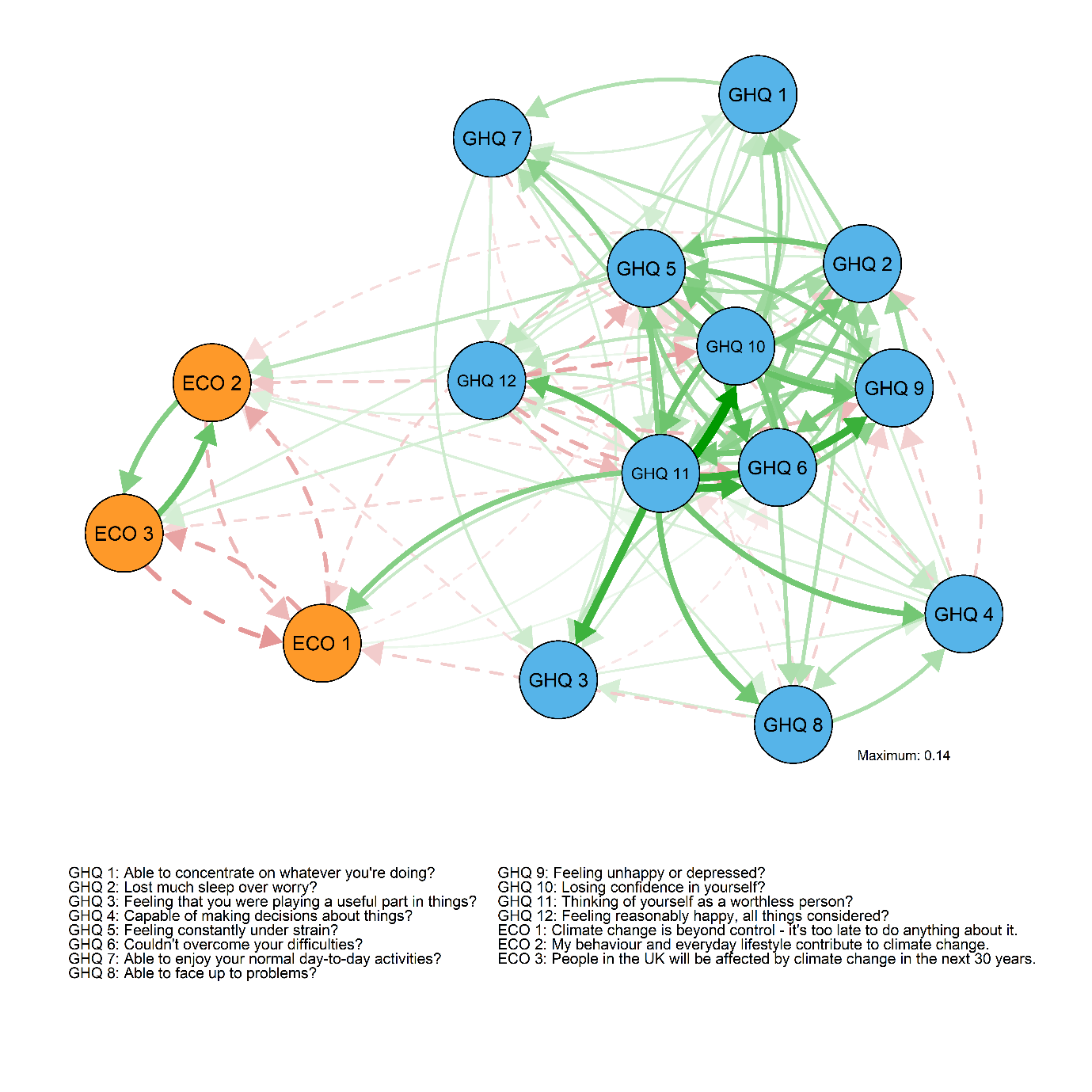


Cross-lagged panel network, autoregressive edges removed. Green/solid lines represent positive and red/dashed lines negative edges. The directed arrows represent cross-lagged associations where the node of origin is a variable measured in wave 4 and the end node is a variable measured in wave 10.

**S5** The edge weights of the cross-lagged panel network with only complete cases

|  | Wave 10 | | | | | | | | | | | | | | |
| --- | --- | --- | --- | --- | --- | --- | --- | --- | --- | --- | --- | --- | --- | --- | --- |
| Wave 4 | GHQ 1 | GHQ 2 | GHQ 3 | GHQ 4 | GHQ 5 | GHQ 6 | GHQ 7 | GHQ 8 | GHQ 9 | GHQ 10 | GHQ 11 | GHQ 12 | ECO 1 | ECO 2 | ECO 3 |
| GHQ1 | 0.084 | 0.000 | 0.025 | 0.000 | 0.024 | 0.000 | 0.042 | 0.023 | 0.019 | 0.026 | 0.025 | 0.025 | 0.000 | 0.000 | 0.000 |
| GHQ 2 | 0.047 | 0.223 | 0.025 | 0.020 | 0.075 | 0.064 | 0.038 | 0.042 | 0.061 | 0.051 | 0.037 | 0.021 | 0.000 | -0.019 | 0.000 |
| GHQ 3 | 0.000 | 0.000 | 0.071 | 0.017 | 0.000 | -0.013 | 0.023 | 0.000 | 0.000 | 0.000 | 0.000 | 0.000 | 0.000 | -0.019 | 0.000 |
| GHQ 4 | 0.022 | -0.028 | 0.000 | 0.083 | -0.026 | 0.000 | 0.000 | 0.038 | -0.025 | 0.000 | -0.015 | 0.024 | 0.000 | 0.020 | 0.000 |
| GHQ 5 | 0.034 | 0.048 | 0.000 | 0.000 | 0.167 | 0.058 | 0.018 | 0.000 | 0.067 | 0.043 | 0.000 | 0.034 | -0.026 | 0.036 | 0.021 |
| GHQ 6 | 0.035 | 0.048 | 0.034 | 0.031 | 0.072 | 0.131 | 0.043 | 0.044 | 0.062 | 0.068 | 0.076 | 0.028 | 0.030 | 0.000 | 0.000 |
| GHQ 7 | 0.021 | 0.000 | 0.026 | 0.000 | 0.000 | 0.000 | 0.081 | 0.000 | 0.000 | -0.027 | -0.016 | 0.022 | 0.000 | 0.000 | 0.000 |
| GHQ 8 | 0.000 | 0.000 | 0.022 | 0.045 | -0.018 | 0.000 | 0.000 | 0.076 | -0.025 | 0.000 | -0.017 | 0.000 | -0.027 | 0.000 | 0.000 |
| GHQ 9 | 0.000 | 0.052 | 0.000 | 0.000 | 0.066 | 0.073 | 0.026 | 0.000 | 0.163 | 0.068 | 0.048 | 0.033 | 0.000 | 0.000 | 0.000 |
| GHQ 10 | 0.031 | 0.075 | 0.000 | 0.025 | 0.067 | 0.093 | 0.019 | 0.000 | 0.082 | 0.192 | 0.079 | 0.000 | 0.000 | 0.000 | 0.023 |
| GHQ 11 | 0.058 | 0.064 | 0.104 | 0.075 | 0.068 | 0.107 | 0.062 | 0.077 | 0.106 | 0.137 | 0.291 | 0.084 | 0.065 | -0.019 | -0.022 |
| GHQ 12 | 0.000 | -0.028 | 0.000 | 0.000 | -0.041 | -0.045 | 0.000 | 0.022 | -0.038 | -0.050 | -0.039 | 0.063 | 0.000 | -0.031 | 0.000 |
| ECO 1 | 0.000 | 0.000 | 0.000 | 0.000 | -0.014 | 0.011 | 0.000 | 0.000 | 0.000 | 0.000 | 0.011 | 0.000 | 0.285 | -0.046 | -0.049 |
| ECO 2 | 0.000 | 0.016 | 0.000 | 0.000 | 0.000 | 0.000 | 0.000 | 0.000 | 0.000 | 0.012 | 0.000 | 0.000 | -0.038 | 0.257 | 0.066 |
| ECO 3 | 0.000 | 0.000 | 0.000 | 0.000 | 0.000 | 0.000 | 0.000 | 0.000 | 0.000 | 0.000 | 0.000 | 0.000 | -0.056 | 0.081 | 0.285 |

Note. The estimates are edge weights of variables measured in wave 4 predicting variables measured in wave 10. Cross-construct associations highlighted. Model fit: χ2(df = 33) = 12.40, p = 1.000, RMSEA = .000 CFI = 1.00, TLI = 1.00. n = 21,632 in the regularized regression step and n = 21,632 in the non-regularized regression step.

**S6** Cross-construct predictability (in) and influence (out) for each node using only complete cases


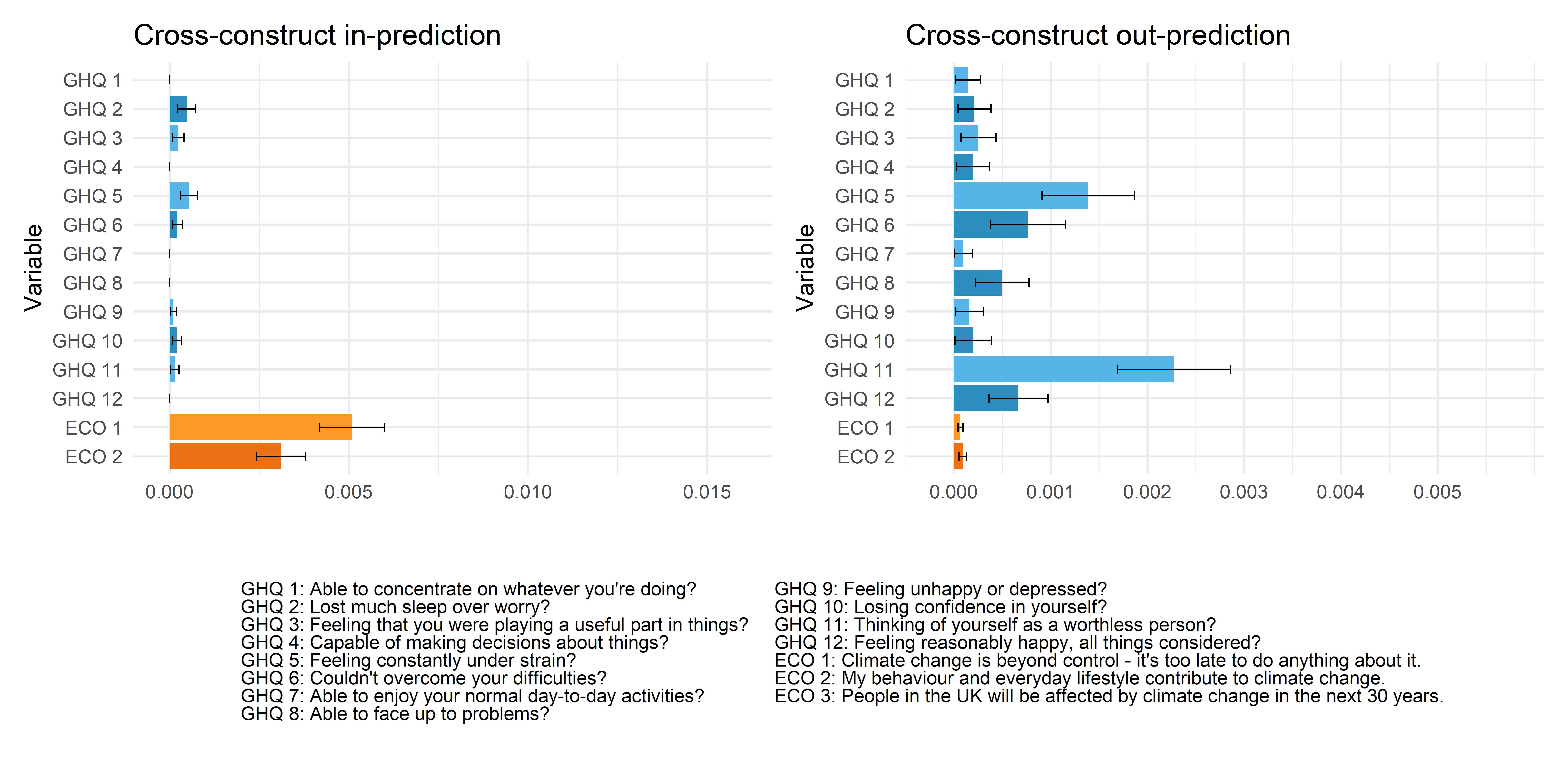


**S7** The cross-lagged panel network model excluding the binary item


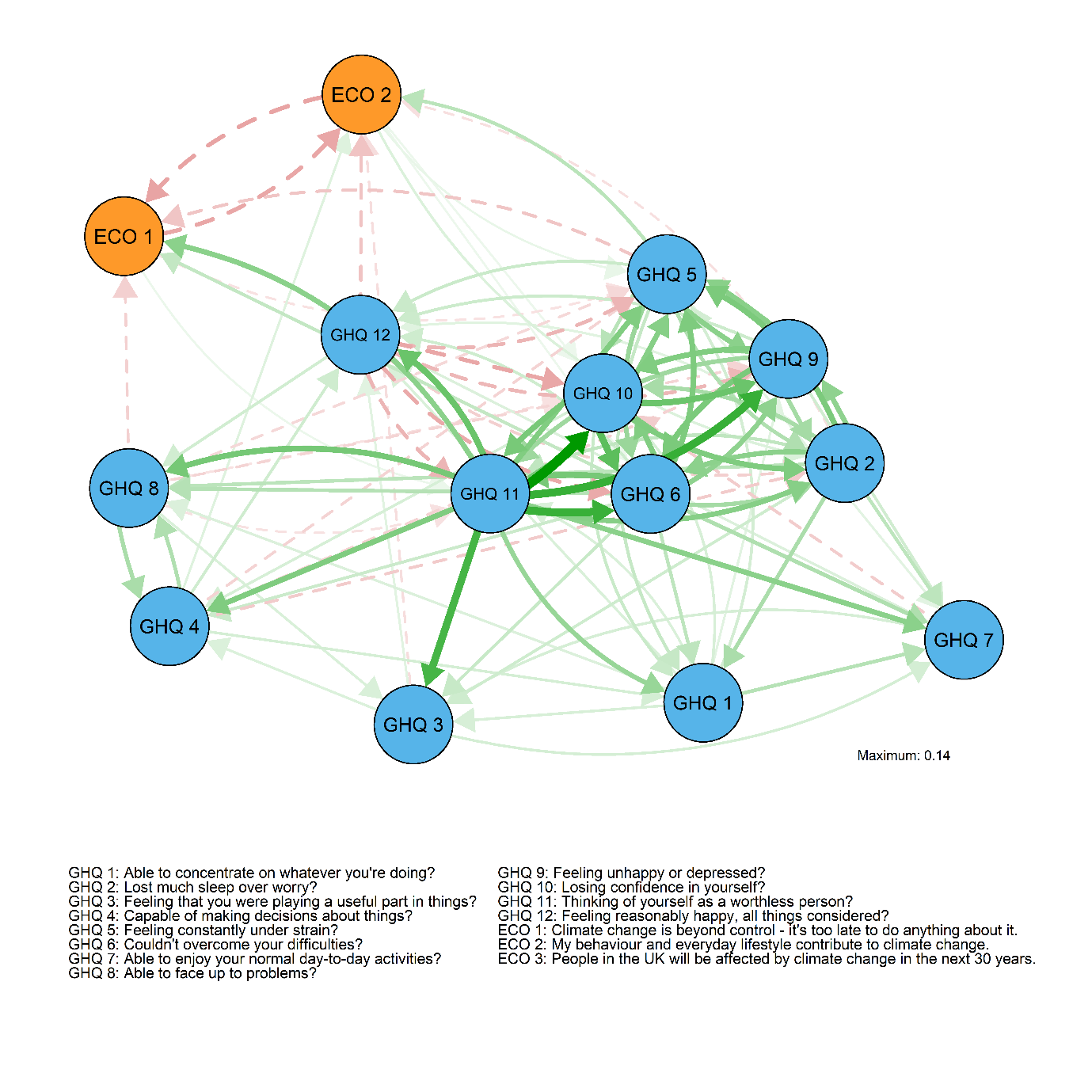


Cross-lagged panel network, autoregressive edges removed. Green/solid lines represent positive and red/dashed lines negative edges. The directed arrows represent cross-lagged associations where the node of origin is a variable measured in wave 4 and the end node is a variable measured in wave 10.

**S8** The edge weights of the cross-lagged panel network excluding the binary item

|  | Wave 10 | | | | | | | | | | | | | |
| --- | --- | --- | --- | --- | --- | --- | --- | --- | --- | --- | --- | --- | --- | --- |
| Wave 4 | GHQ 1 | GHQ 2 | GHQ 3 | GHQ 4 | GHQ 5 | GHQ 6 | GHQ 7 | GHQ 8 | GHQ 9 | GHQ 10 | GHQ 11 | GHQ 12 | ECO 1 | ECO 2 |
| GHQ1 | 0.088 | 0.000 | 0.023 | 0.000 | 0.025 | 0.000 | 0.041 | 0.020 | 0.017 | 0.023 | 0.022 | 0.021 | 0.000 | 0.000 |
| GHQ 2 | 0.047 | 0.228 | 0.027 | 0.000 | 0.074 | 0.064 | 0.037 | 0.038 | 0.063 | 0.049 | 0.035 | 0.017 | 0.000 | -0.018 |
| GHQ 3 | 0.000 | 0.000 | 0.079 | 0.020 | 0.000 | 0.000 | 0.024 | 0.000 | 0.000 | 0.000 | 0.000 | 0.017 | 0.000 | -0.019 |
| GHQ 4 | 0.024 | -0.029 | 0.000 | 0.089 | -0.025 | 0.000 | 0.000 | 0.045 | -0.025 | 0.000 | 0.000 | 0.027 | 0.000 | 0.018 |
| GHQ 5 | 0.032 | 0.044 | 0.000 | 0.000 | 0.168 | 0.057 | 0.016 | 0.000 | 0.065 | 0.040 | 0.000 | 0.030 | -0.033 | 0.038 |
| GHQ 6 | 0.037 | 0.050 | 0.031 | 0.029 | 0.072 | 0.134 | 0.045 | 0.042 | 0.063 | 0.070 | 0.077 | 0.027 | 0.034 | 0.000 |
| GHQ 7 | 0.000 | 0.000 | 0.024 | 0.000 | 0.000 | 0.000 | 0.078 | 0.000 | 0.000 | -0.028 | 0.000 | 0.020 | 0.000 | 0.000 |
| GHQ 8 | 0.000 | 0.000 | 0.022 | 0.048 | -0.022 | 0.000 | 0.000 | 0.080 | -0.024 | -0.017 | -0.017 | 0.000 | -0.026 | 0.000 |
| GHQ 9 | 0.000 | 0.051 | 0.000 | 0.000 | 0.066 | 0.074 | 0.026 | 0.000 | 0.166 | 0.071 | 0.054 | 0.028 | 0.000 | 0.000 |
| GHQ 10 | 0.030 | 0.072 | 0.000 | 0.023 | 0.067 | 0.092 | 0.000 | 0.000 | 0.083 | 0.194 | 0.076 | 0.000 | 0.000 | 0.000 |
| GHQ 11 | 0.058 | 0.067 | 0.104 | 0.071 | 0.071 | 0.111 | 0.065 | 0.077 | 0.113 | 0.143 | 0.300 | 0.083 | 0.065 | 0.000 |
| GHQ 12 | 0.000 | -0.027 | 0.000 | 0.000 | -0.041 | -0.048 | 0.000 | 0.026 | -0.040 | -0.049 | -0.042 | 0.068 | 0.000 | -0.034 |
| ECO 1 | 0.000 | 0.000 | 0.000 | 0.000 | -0.017 | 0.000 | 0.000 | 0.000 | 0.000 | 0.000 | 0.011 | 0.000 | 0.292 | -0.050 |
| ECO 2 | 0.000 | 0.020 | 0.000 | 0.000 | 0.013 | 0.011 | 0.000 | 0.000 | 0.000 | 0.011 | 0.000 | 0.000 | -0.051 | 0.275 |

Note. The estimates are edge weights of variables measured in wave 4 predicting variables measured in wave 10. Cross-construct associations highlighted. Model fit: χ2(df = 21) = 15.21, p = 0.812, RMSEA = .000 CFI = 1.00, TLI = 1.00. n = 21,632 in the regularized regression step and n = 34,318 in the non-regularized regression step.

**S9** Cross-construct predictability (in) and influence (out) for each node of the network model excluding the binary item

**
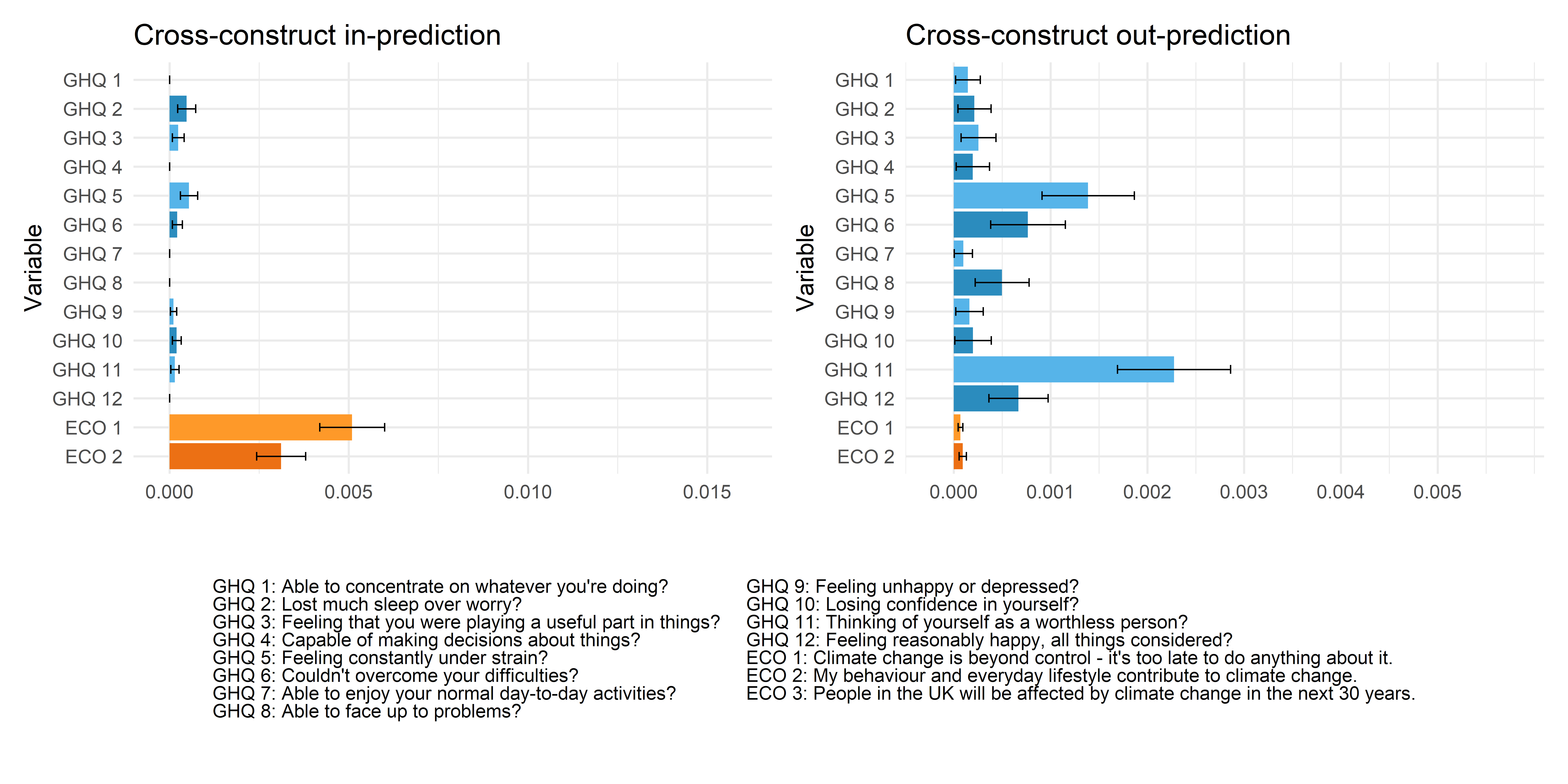
**

**S10** The cross-lagged panel network models for men and women


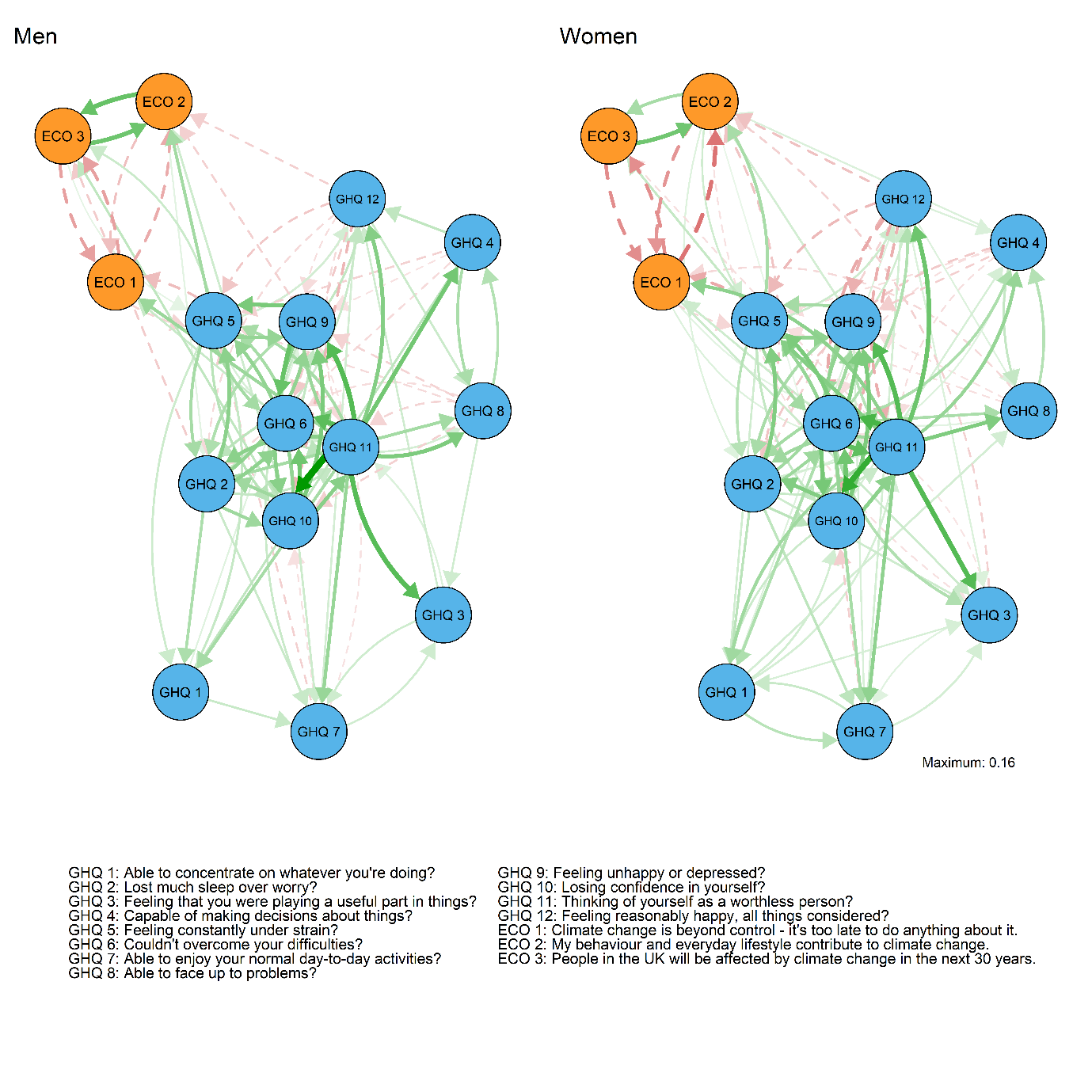


Cross-lagged panel network, autoregressive edges removed. Green/solid lines represent positive and red/dashed lines negative edges. The directed arrows represent cross-lagged associations where the node of origin is a variable measured in wave 4 and the end node is a variable measured in wave 10.

**S11** The edge weights of the cross-lagged panel network for men

|  | Wave 10 | | | | | | | | | | | | | | |
| --- | --- | --- | --- | --- | --- | --- | --- | --- | --- | --- | --- | --- | --- | --- | --- |
| Wave 4 | GHQ 1 | GHQ 2 | GHQ 3 | GHQ 4 | GHQ 5 | GHQ 6 | GHQ 7 | GHQ 8 | GHQ 9 | GHQ 10 | GHQ 11 | GHQ 12 | ECO 1 | ECO 2 | ECO 3 |
| GHQ1 | 0.086 | 0.000 | 0.000 | 0.000 | 0.036 | 0.000 | 0.035 | 0.000 | 0.021 | 0.000 | 0.000 | 0.000 | 0.000 | 0.000 | 0.000 |
| GHQ 2 | 0.052 | 0.227 | 0.000 | 0.000 | 0.071 | 0.069 | 0.034 | 0.056 | 0.067 | 0.060 | 0.037 | 0.033 | 0.000 | 0.000 | -0.032 |
| GHQ 3 | 0.000 | 0.000 | 0.080 | 0.000 | 0.000 | 0.000 | 0.030 | 0.000 | 0.000 | 0.000 | 0.022 | 0.031 | 0.000 | 0.000 | 0.000 |
| GHQ 4 | 0.040 | -0.023 | 0.000 | 0.096 | -0.029 | 0.000 | 0.000 | 0.052 | -0.018 | 0.000 | 0.000 | 0.039 | 0.000 | 0.000 | 0.000 |
| GHQ 5 | 0.031 | 0.055 | 0.000 | 0.000 | 0.187 | 0.056 | 0.000 | 0.000 | 0.065 | 0.044 | 0.025 | 0.027 | -0.041 | 0.057 | 0.033 |
| GHQ 6 | 0.000 | 0.041 | 0.000 | 0.000 | 0.055 | 0.102 | 0.031 | 0.027 | 0.037 | 0.046 | 0.042 | 0.000 | 0.034 | 0.000 | 0.000 |
| GHQ 7 | 0.000 | 0.000 | 0.030 | 0.000 | -0.030 | 0.000 | 0.066 | 0.000 | -0.019 | -0.019 | 0.000 | 0.000 | 0.000 | 0.000 | 0.000 |
| GHQ 8 | 0.000 | 0.000 | 0.030 | 0.047 | -0.024 | -0.022 | 0.000 | 0.070 | -0.027 | -0.029 | -0.027 | 0.000 | 0.000 | 0.000 | 0.000 |
| GHQ 9 | 0.000 | 0.050 | 0.000 | 0.000 | 0.082 | 0.093 | 0.034 | 0.000 | 0.180 | 0.073 | 0.062 | 0.038 | 0.000 | -0.029 | 0.000 |
| GHQ 10 | 0.000 | 0.060 | 0.000 | 0.000 | 0.067 | 0.088 | 0.000 | 0.000 | 0.081 | 0.187 | 0.074 | 0.000 | 0.000 | 0.031 | 0.039 |
| GHQ 11 | 0.055 | 0.072 | 0.103 | 0.087 | 0.055 | 0.102 | 0.064 | 0.080 | 0.105 | 0.158 | 0.294 | 0.075 | 0.062 | 0.000 | 0.000 |
| GHQ 12 | 0.000 | -0.024 | 0.000 | 0.000 | -0.035 | -0.033 | 0.027 | 0.033 | -0.021 | -0.045 | -0.024 | 0.071 | 0.000 | -0.030 | 0.000 |
| ECO 1 | 0.000 | 0.000 | 0.000 | 0.000 | -0.027 | 0.000 | 0.000 | 0.000 | 0.000 | 0.000 | 0.000 | 0.000 | 0.293 | -0.052 | -0.059 |
| ECO 2 | 0.000 | 0.018 | 0.000 | 0.000 | 0.000 | 0.000 | 0.000 | 0.000 | 0.000 | 0.000 | 0.000 | 0.000 | -0.039 | 0.309 | 0.094 |
| ECO 3 | 0.000 | 0.000 | 0.000 | 0.000 | 0.017 | 0.000 | 0.000 | 0.000 | 0.000 | 0.000 | 0.000 | 0.000 | -0.058 | 0.090 | 0.310 |

Note. The estimates are edge weights of variables measured in wave 4 predicting variables measured in wave 10. Cross-construct associations highlighted. Model fit: χ2(df = 51) = 16.89, p = 1.000, RMSEA = .000 CFI = 1.00, TLI = 1.00. n = 9,370 in the regularized regression step and n = 11,592 in the non-regularized regression step.

**S12** The edge weights of the cross-lagged panel network excluding the binary item for women

|  | Wave 10 | | | | | | | | | | | | | | |
| --- | --- | --- | --- | --- | --- | --- | --- | --- | --- | --- | --- | --- | --- | --- | --- |
| Wave 4 | GHQ 1 | GHQ 2 | GHQ 3 | GHQ 4 | GHQ 5 | GHQ 6 | GHQ 7 | GHQ 8 | GHQ 9 | GHQ 10 | GHQ 11 | GHQ 12 | ECO 1 | ECO 2 | ECO 3 |
| GHQ1 | 0.081 | 0.000 | 0.025 | 0.000 | 0.000 | 0.000 | 0.044 | 0.027 | 0.000 | 0.028 | 0.027 | 0.022 | 0.000 | 0.000 | 0.000 |
| GHQ 2 | 0.043 | 0.222 | 0.031 | 0.021 | 0.077 | 0.061 | 0.041 | 0.027 | 0.059 | 0.041 | 0.034 | 0.000 | 0.000 | -0.037 | 0.000 |
| GHQ 3 | 0.000 | 0.000 | 0.074 | 0.000 | 0.000 | -0.018 | 0.020 | 0.000 | 0.000 | 0.000 | 0.000 | 0.000 | 0.000 | -0.029 | 0.000 |
| GHQ 4 | 0.000 | -0.034 | 0.000 | 0.075 | -0.021 | -0.020 | 0.000 | 0.037 | -0.030 | 0.000 | -0.021 | 0.000 | 0.000 | 0.023 | 0.000 |
| GHQ 5 | 0.030 | 0.045 | 0.000 | 0.000 | 0.161 | 0.057 | 0.000 | 0.000 | 0.068 | 0.037 | 0.000 | 0.031 | -0.053 | 0.054 | 0.036 |
| GHQ 6 | 0.050 | 0.056 | 0.049 | 0.052 | 0.081 | 0.152 | 0.055 | 0.053 | 0.079 | 0.084 | 0.099 | 0.049 | 0.031 | 0.000 | 0.000 |
| GHQ 7 | 0.034 | 0.000 | 0.027 | 0.000 | 0.000 | 0.000 | 0.088 | 0.000 | 0.000 | -0.030 | 0.000 | 0.027 | 0.000 | 0.000 | 0.000 |
| GHQ 8 | 0.000 | 0.000 | 0.000 | 0.046 | -0.020 | 0.000 | 0.000 | 0.077 | -0.019 | 0.000 | 0.000 | 0.000 | -0.024 | 0.000 | 0.000 |
| GHQ 9 | 0.000 | 0.053 | 0.000 | 0.000 | 0.056 | 0.061 | 0.025 | 0.000 | 0.154 | 0.068 | 0.045 | 0.029 | 0.000 | 0.028 | 0.000 |
| GHQ 10 | 0.033 | 0.077 | 0.000 | 0.026 | 0.065 | 0.089 | 0.000 | 0.000 | 0.080 | 0.192 | 0.071 | 0.000 | 0.000 | 0.000 | 0.000 |
| GHQ 11 | 0.059 | 0.060 | 0.106 | 0.065 | 0.075 | 0.114 | 0.069 | 0.078 | 0.105 | 0.126 | 0.295 | 0.090 | 0.070 | 0.000 | -0.021 |
| GHQ 12 | 0.000 | -0.027 | 0.022 | 0.023 | -0.043 | -0.053 | 0.000 | 0.022 | -0.049 | -0.049 | -0.050 | 0.060 | 0.000 | -0.038 | 0.000 |
| ECO 1 | 0.000 | 0.000 | 0.032 | 0.000 | -0.026 | 0.000 | 0.000 | 0.019 | 0.000 | 0.000 | 0.022 | 0.000 | 0.315 | -0.086 | -0.070 |
| ECO 2 | 0.000 | 0.021 | -0.019 | 0.000 | 0.030 | 0.000 | 0.000 | 0.000 | 0.000 | 0.014 | 0.000 | 0.000 | -0.065 | 0.228 | 0.054 |
| ECO 3 | 0.000 | 0.000 | 0.000 | 0.000 | 0.000 | 0.000 | 0.000 | 0.000 | 0.000 | 0.000 | 0.000 | 0.000 | -0.070 | 0.088 | 0.277 |

Note. The estimates are edge weights of variables measured in wave 4 predicting variables measured in wave 10. Cross-construct associations highlighted. Model fit: χ2(df = 38) = 15.58, p = 1.000, RMSEA = .000 CFI = 1.00, TLI = 1.00. n = 12,314 in the regularized regression step and n = 14,711 in the non-regularized regression step.

**S13** Cross-construct predictability (in) and influence (out) for each node for men vs. women


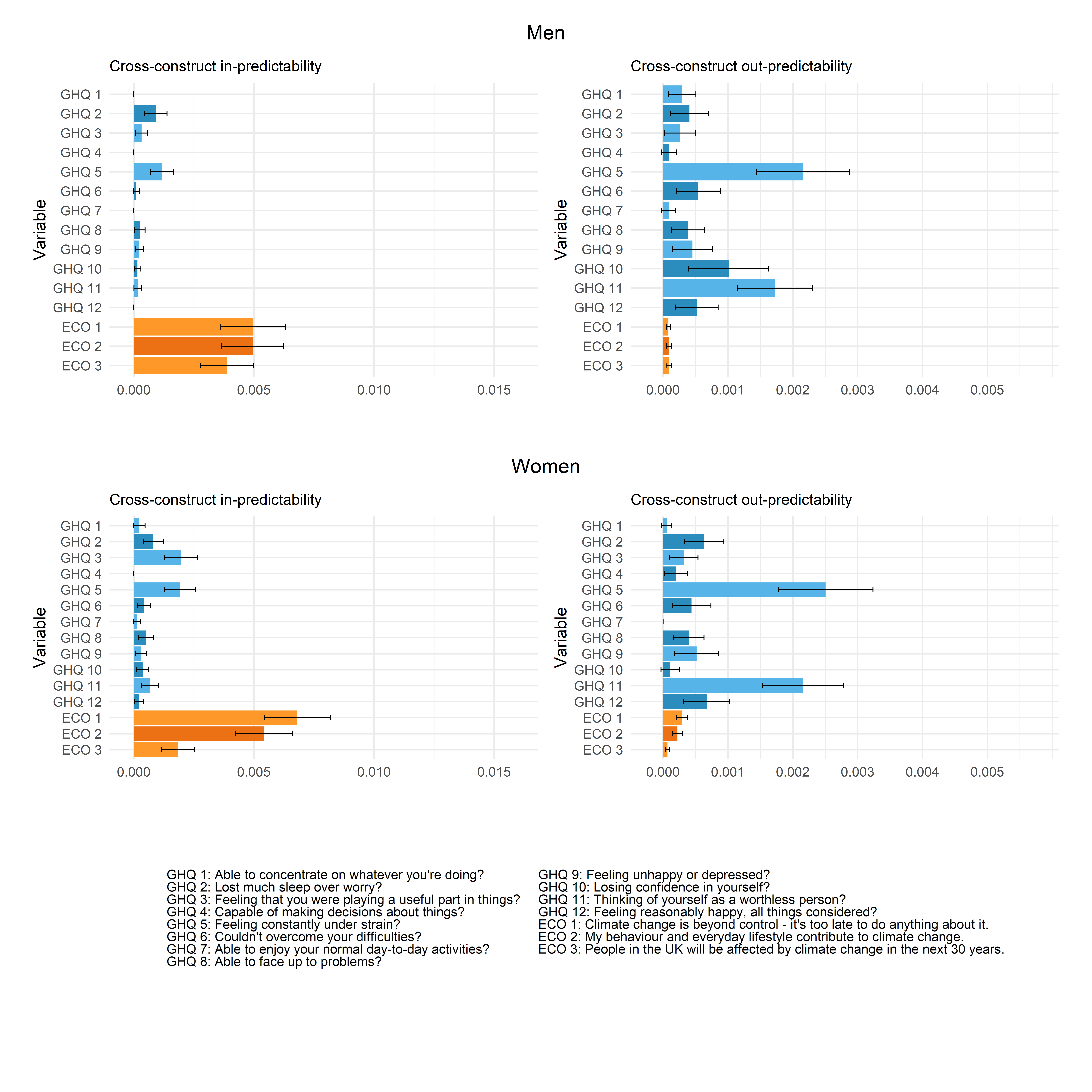


**S14** The cross-lagged panel network models for high and low education groups


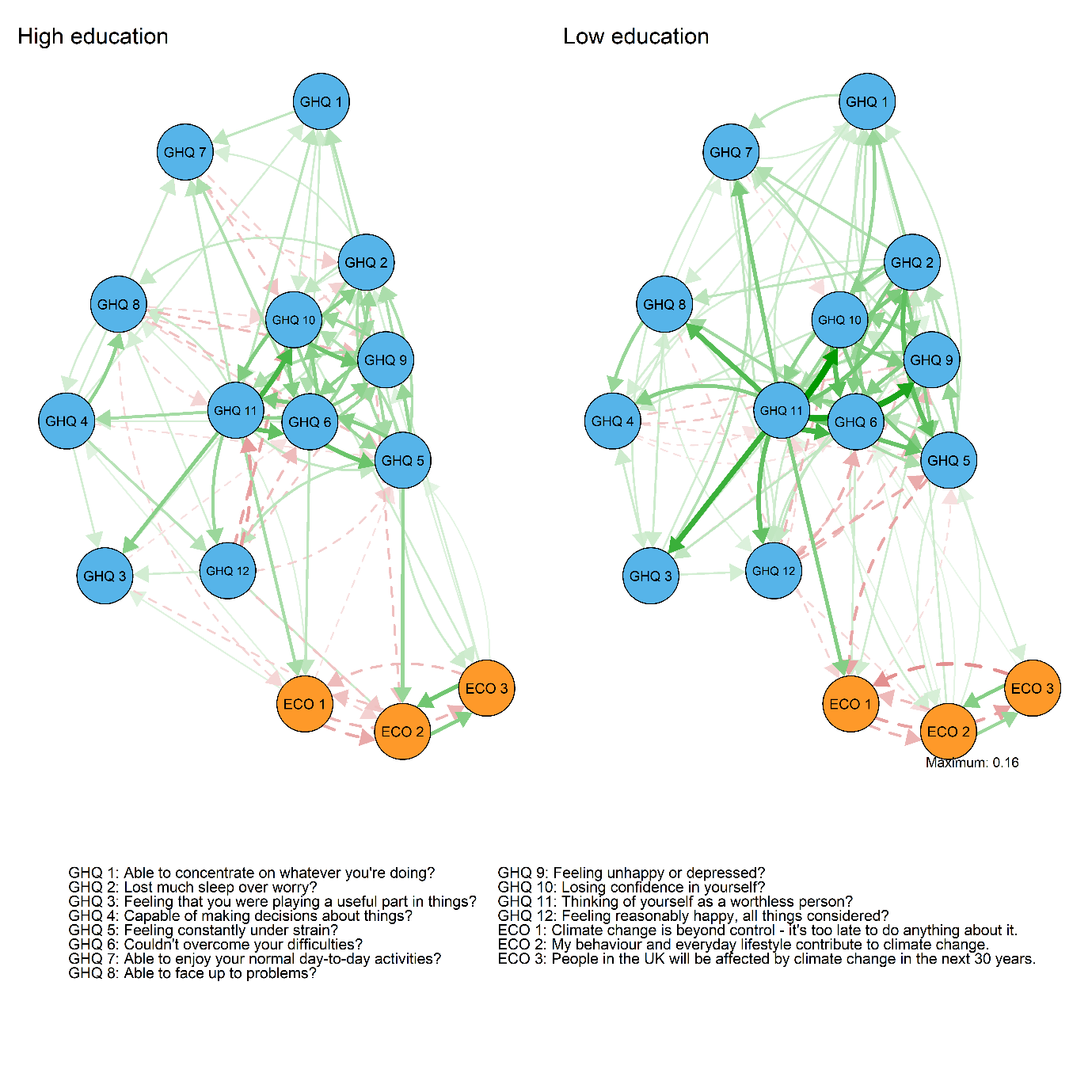


Cross-lagged panel network, autoregressive edges removed. Green/solid lines represent positive and red/dashed lines negative edges. The directed arrows represent cross-lagged associations where the node of origin is a variable measured in wave 4 and the end node is a variable measured in wave 10.

**S15** The edge weights of the cross-lagged panel network for the high education group

|  | Wave 10 | | | | | | | | | | | | | | |
| --- | --- | --- | --- | --- | --- | --- | --- | --- | --- | --- | --- | --- | --- | --- | --- |
| Wave 4 | GHQ 1 | GHQ 2 | GHQ 3 | GHQ 4 | GHQ 5 | GHQ 6 | GHQ 7 | GHQ 8 | GHQ 9 | GHQ 10 | GHQ 11 | GHQ 12 | ECO 1 | ECO 2 | ECO 3 |
| GHQ1 | 0.084 | 0.000 | 0.000 | 0.000 | 0.000 | 0.000 | 0.039 | 0.000 | 0.000 | 0.029 | 0.000 | 0.000 | 0.000 | 0.000 | 0.000 |
| GHQ 2 | 0.046 | 0.222 | 0.025 | 0.000 | 0.053 | 0.051 | 0.027 | 0.035 | 0.056 | 0.038 | 0.023 | 0.000 | 0.000 | -0.040 | 0.000 |
| GHQ 3 | 0.000 | 0.000 | 0.049 | 0.000 | 0.000 | -0.019 | 0.000 | 0.000 | 0.000 | 0.000 | 0.000 | 0.000 | 0.000 | -0.029 | 0.000 |
| GHQ 4 | 0.026 | 0.000 | 0.028 | 0.098 | -0.022 | 0.000 | 0.031 | 0.069 | 0.000 | 0.000 | 0.000 | 0.045 | 0.000 | 0.033 | 0.000 |
| GHQ 5 | 0.035 | 0.051 | 0.000 | 0.000 | 0.197 | 0.075 | 0.000 | 0.000 | 0.066 | 0.051 | 0.025 | 0.041 | 0.000 | 0.064 | 0.032 |
| GHQ 6 | 0.031 | 0.064 | 0.000 | 0.027 | 0.092 | 0.146 | 0.050 | 0.000 | 0.062 | 0.073 | 0.083 | 0.000 | 0.041 | 0.000 | 0.000 |
| GHQ 7 | 0.000 | -0.030 | 0.000 | 0.000 | 0.000 | 0.000 | 0.076 | 0.000 | -0.027 | -0.037 | 0.000 | 0.000 | 0.000 | 0.000 | 0.000 |
| GHQ 8 | 0.000 | -0.029 | 0.000 | 0.028 | -0.039 | -0.027 | -0.030 | 0.064 | -0.043 | -0.027 | -0.026 | 0.000 | -0.026 | 0.000 | 0.000 |
| GHQ 9 | 0.000 | 0.053 | 0.000 | 0.000 | 0.058 | 0.070 | 0.000 | 0.000 | 0.170 | 0.065 | 0.052 | 0.000 | 0.000 | 0.000 | 0.000 |
| GHQ 10 | 0.000 | 0.074 | 0.000 | 0.000 | 0.062 | 0.078 | 0.000 | 0.000 | 0.090 | 0.203 | 0.080 | 0.000 | 0.000 | 0.000 | 0.030 |
| GHQ 11 | 0.041 | 0.033 | 0.074 | 0.057 | 0.038 | 0.098 | 0.041 | 0.033 | 0.071 | 0.114 | 0.264 | 0.062 | 0.054 | 0.000 | 0.000 |
| GHQ 12 | 0.000 | 0.000 | 0.028 | 0.000 | -0.030 | -0.042 | 0.000 | 0.027 | -0.034 | -0.057 | -0.061 | 0.079 | 0.000 | -0.036 | 0.000 |
| ECO 1 | 0.000 | 0.000 | 0.020 | 0.019 | -0.020 | 0.000 | 0.000 | 0.021 | 0.000 | 0.000 | 0.000 | 0.000 | 0.327 | -0.054 | -0.047 |
| ECO 2 | 0.000 | 0.000 | 0.000 | 0.000 | 0.000 | 0.000 | 0.000 | 0.000 | 0.000 | 0.000 | 0.000 | 0.000 | -0.039 | 0.298 | 0.076 |
| ECO 3 | 0.000 | 0.027 | 0.000 | 0.000 | 0.018 | 0.000 | 0.000 | 0.000 | 0.022 | 0.000 | 0.000 | 0.000 | -0.042 | 0.089 | 0.322 |

Note. The estimates are edge weights of variables measured in wave 4 predicting variables measured in wave 10. Cross-construct associations highlighted. Model fit: χ2(df = 39) = 14.50, p = 1.000, RMSEA = .000 CFI = 1.00, TLI = 1.00. n = 9,705 in the regularized regression step and n = 13,573 in the non-regularized regression step.

**S16** The edge weights of the cross-lagged panel network for the low education group

|  | Wave 10 | | | | | | | | | | | | | | |
| --- | --- | --- | --- | --- | --- | --- | --- | --- | --- | --- | --- | --- | --- | --- | --- |
| Wave 4 | GHQ 1 | GHQ 2 | GHQ 3 | GHQ 4 | GHQ 5 | GHQ 6 | GHQ 7 | GHQ 8 | GHQ 9 | GHQ 10 | GHQ 11 | GHQ 12 | ECO 1 | ECO 2 | ECO 3 |
| GHQ1 | 0.090 | 0.000 | 0.000 | 0.000 | 0.029 | 0.000 | 0.042 | 0.026 | 0.020 | 0.000 | 0.032 | 0.000 | 0.000 | 0.000 | 0.000 |
| GHQ 2 | 0.054 | 0.246 | 0.031 | 0.027 | 0.101 | 0.079 | 0.050 | 0.043 | 0.074 | 0.071 | 0.049 | 0.031 | 0.000 | 0.000 | 0.000 |
| GHQ 3 | 0.028 | 0.000 | 0.102 | 0.031 | 0.000 | 0.000 | 0.047 | 0.000 | -0.017 | 0.000 | 0.000 | 0.027 | 0.000 | 0.000 | 0.000 |
| GHQ 4 | 0.023 | -0.034 | 0.000 | 0.088 | -0.025 | -0.019 | 0.000 | 0.028 | -0.033 | 0.000 | -0.019 | 0.000 | 0.000 | 0.000 | 0.000 |
| GHQ 5 | 0.030 | 0.045 | 0.000 | 0.000 | 0.148 | 0.042 | 0.000 | 0.000 | 0.068 | 0.034 | 0.000 | 0.000 | -0.057 | 0.025 | 0.026 |
| GHQ 6 | 0.040 | 0.038 | 0.039 | 0.030 | 0.053 | 0.126 | 0.042 | 0.059 | 0.063 | 0.065 | 0.074 | 0.035 | 0.030 | 0.029 | 0.000 |
| GHQ 7 | 0.024 | 0.000 | 0.029 | 0.000 | 0.000 | 0.023 | 0.081 | 0.000 | 0.000 | -0.020 | 0.000 | 0.039 | 0.000 | 0.000 | 0.000 |
| GHQ 8 | 0.000 | 0.000 | 0.032 | 0.064 | 0.000 | 0.000 | 0.021 | 0.091 | 0.000 | 0.000 | 0.000 | 0.022 | -0.027 | 0.000 | 0.000 |
| GHQ 9 | 0.000 | 0.043 | 0.000 | 0.000 | 0.071 | 0.077 | 0.036 | 0.000 | 0.159 | 0.071 | 0.052 | 0.037 | 0.029 | 0.000 | 0.000 |
| GHQ 10 | 0.037 | 0.082 | 0.000 | 0.034 | 0.078 | 0.103 | 0.000 | 0.000 | 0.079 | 0.196 | 0.071 | 0.000 | 0.000 | 0.000 | 0.000 |
| GHQ 11 | 0.064 | 0.089 | 0.123 | 0.077 | 0.094 | 0.117 | 0.081 | 0.107 | 0.142 | 0.160 | 0.321 | 0.098 | 0.076 | 0.000 | 0.000 |
| GHQ 12 | 0.000 | -0.036 | 0.000 | 0.000 | -0.052 | -0.050 | 0.000 | 0.027 | -0.044 | -0.044 | -0.026 | 0.061 | 0.000 | -0.024 | 0.000 |
| ECO 1 | 0.000 | 0.000 | 0.000 | 0.000 | -0.020 | 0.000 | 0.000 | 0.000 | 0.000 | 0.000 | 0.000 | 0.000 | 0.272 | -0.047 | -0.058 |
| ECO 2 | 0.000 | 0.030 | 0.000 | 0.000 | 0.021 | 0.017 | 0.000 | 0.000 | 0.000 | 0.016 | 0.016 | 0.000 | -0.043 | 0.227 | 0.063 |
| ECO 3 | 0.000 | 0.000 | 0.000 | 0.000 | 0.000 | 0.014 | 0.000 | 0.000 | 0.000 | 0.000 | 0.000 | 0.000 | -0.069 | 0.076 | 0.273 |

Note. The estimates are edge weights of variables measured in wave 4 predicting variables measured in wave 10. Cross-construct associations highlighted. Model fit: χ2(df = 47) = 19.20, p = 1.000, RMSEA = .000 CFI = 1.00, TLI = 1.00. n = 11,946 in the regularized regression step and n = 20,120 in the non-regularized regression step.

**S17** Cross-construct predictability (in) and influence (out) for each node in low vs. high education groups


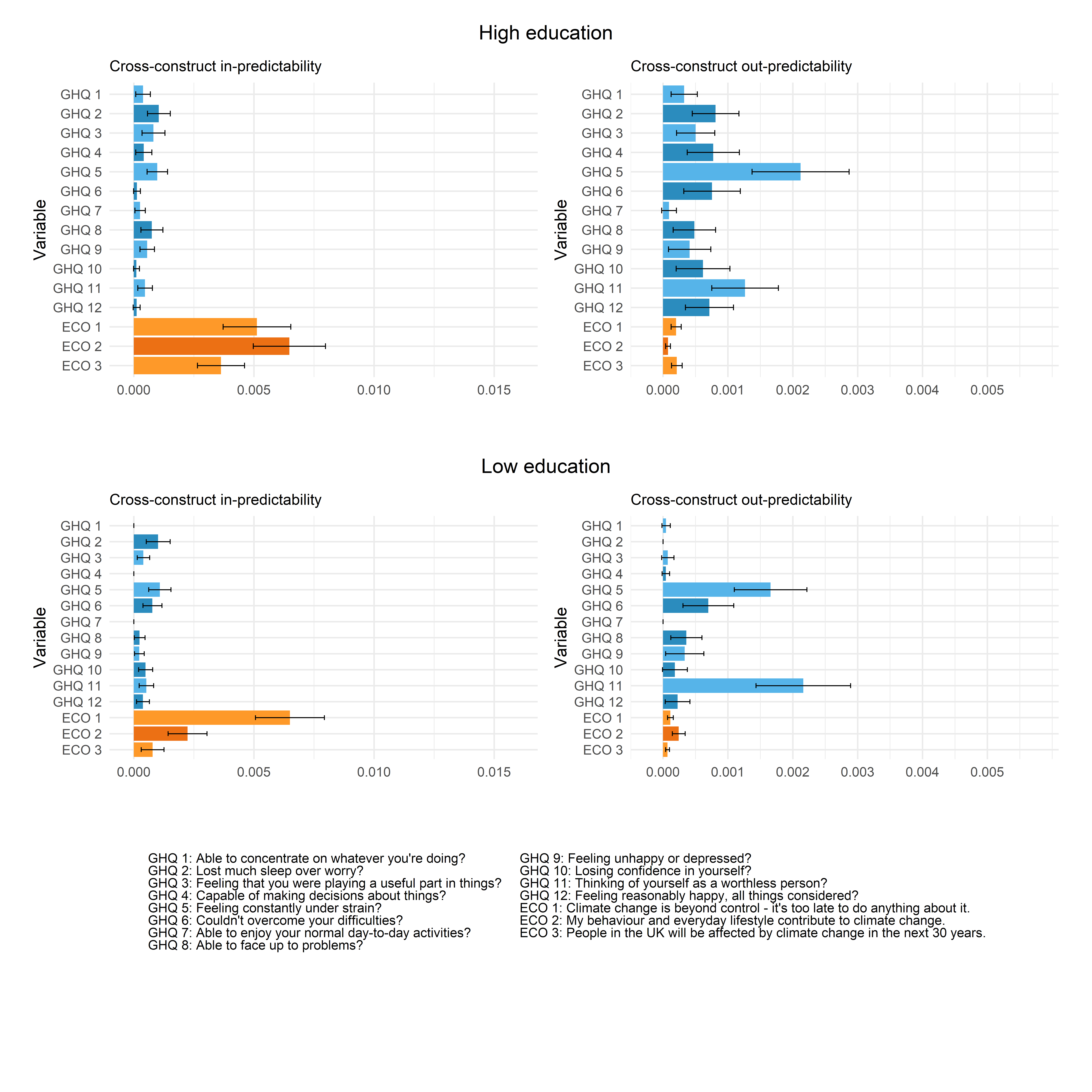


**S18** The edge weights of the cross-lagged panel network for the high internal political efficacy group

|  | Wave 10 | | | | | | | | | | | | | | |
| --- | --- | --- | --- | --- | --- | --- | --- | --- | --- | --- | --- | --- | --- | --- | --- |
| Wave 4 | GHQ 1 | GHQ 2 | GHQ 3 | GHQ 4 | GHQ 5 | GHQ 6 | GHQ 7 | GHQ 8 | GHQ 9 | GHQ 10 | GHQ 11 | GHQ 12 | ECO 1 | ECO 2 | ECO 3 |
| GHQ1 | 0.084 | 0.000 | 0.027 | 0.000 | 0.000 | 0.000 | 0.041 | 0.000 | 0.023 | 0.023 | 0.000 | 0.023 | 0.000 | 0.020 | 0.000 |
| GHQ 2 | 0.040 | 0.217 | 0.039 | 0.000 | 0.069 | 0.064 | 0.038 | 0.037 | 0.059 | 0.046 | 0.037 | 0.025 | 0.000 | 0.000 | 0.000 |
| GHQ 3 | 0.000 | 0.000 | 0.077 | 0.021 | 0.000 | 0.000 | 0.023 | 0.000 | 0.000 | 0.000 | 0.000 | 0.028 | 0.000 | 0.000 | 0.000 |
| GHQ 4 | 0.000 | -0.022 | 0.000 | 0.082 | 0.000 | 0.000 | 0.000 | 0.041 | -0.022 | 0.000 | -0.019 | 0.026 | 0.000 | 0.000 | 0.000 |
| GHQ 5 | 0.039 | 0.060 | 0.000 | 0.000 | 0.190 | 0.058 | 0.025 | 0.000 | 0.070 | 0.051 | 0.022 | 0.036 | -0.033 | 0.042 | 0.000 |
| GHQ 6 | 0.049 | 0.060 | 0.000 | 0.033 | 0.079 | 0.144 | 0.045 | 0.048 | 0.069 | 0.075 | 0.077 | 0.027 | 0.039 | 0.000 | 0.000 |
| GHQ 7 | 0.000 | 0.000 | 0.024 | 0.000 | 0.000 | 0.000 | 0.066 | 0.000 | -0.017 | -0.020 | 0.000 | 0.000 | 0.000 | 0.000 | 0.000 |
| GHQ 8 | 0.000 | 0.000 | 0.028 | 0.049 | 0.000 | 0.000 | 0.000 | 0.084 | -0.025 | -0.028 | 0.000 | 0.000 | -0.035 | 0.000 | 0.000 |
| GHQ 9 | 0.000 | 0.046 | 0.000 | 0.000 | 0.046 | 0.073 | 0.000 | 0.000 | 0.164 | 0.059 | 0.036 | 0.025 | 0.000 | 0.000 | 0.000 |
| GHQ 10 | 0.026 | 0.065 | 0.000 | 0.000 | 0.070 | 0.090 | 0.000 | 0.000 | 0.081 | 0.193 | 0.084 | 0.000 | 0.000 | 0.023 | 0.000 |
| GHQ 11 | 0.056 | 0.053 | 0.096 | 0.074 | 0.056 | 0.094 | 0.064 | 0.080 | 0.090 | 0.135 | 0.281 | 0.065 | 0.063 | 0.000 | -0.028 |
| GHQ 12 | 0.000 | -0.030 | 0.000 | 0.000 | -0.046 | -0.051 | 0.000 | 0.000 | -0.037 | -0.056 | -0.041 | 0.066 | 0.000 | -0.035 | 0.000 |
| ECO 1 | 0.000 | 0.000 | 0.000 | 0.000 | 0.000 | 0.000 | 0.000 | 0.000 | 0.000 | 0.000 | 0.000 | 0.000 | 0.295 | -0.051 | -0.044 |
| ECO 2 | 0.000 | 0.015 | 0.000 | 0.000 | 0.015 | 0.000 | 0.000 | 0.000 | 0.000 | 0.015 | 0.000 | 0.000 | -0.029 | 0.282 | 0.073 |
| ECO 3 | 0.016 | 0.030 | 0.000 | 0.000 | 0.000 | 0.019 | 0.000 | 0.000 | 0.018 | 0.000 | 0.000 | 0.000 | -0.046 | 0.087 | 0.313 |

Note. The estimates are edge weights of variables measured in wave 4 predicting variables measured in wave 10. Cross-construct associations highlighted. Model fit: χ2(df = 40) = 11.13, p = 1.000, RMSEA = .000 CFI = 1.00, TLI = 1.00. n = 13,317 in the regularized regression step and n = 15,676 in the non-regularized regression step.

**S19** The edge weights of the cross-lagged panel network for the low internal political efficacy group

|  | Wave 10 | | | | | | | | | | | | | | |
| --- | --- | --- | --- | --- | --- | --- | --- | --- | --- | --- | --- | --- | --- | --- | --- |
| Wave 4 | GHQ 1 | GHQ 2 | GHQ 3 | GHQ 4 | GHQ 5 | GHQ 6 | GHQ 7 | GHQ 8 | GHQ 9 | GHQ 10 | GHQ 11 | GHQ 12 | ECO 1 | ECO 2 | ECO 3 |
| GHQ1 | 0.070 | 0.000 | 0.000 | 0.000 | 0.034 | 0.000 | 0.029 | 0.000 | 0.000 | 0.000 | 0.023 | 0.023 | 0.000 | 0.000 | 0.000 |
| GHQ 2 | 0.057 | 0.238 | 0.000 | 0.000 | 0.086 | 0.072 | 0.029 | 0.044 | 0.068 | 0.057 | 0.041 | 0.000 | 0.000 | 0.000 | 0.000 |
| GHQ 3 | 0.031 | 0.000 | 0.074 | 0.027 | 0.000 | 0.000 | 0.040 | 0.000 | 0.000 | 0.000 | 0.000 | 0.000 | -0.029 | 0.000 | 0.000 |
| GHQ 4 | 0.000 | -0.040 | 0.037 | 0.112 | -0.034 | 0.000 | 0.000 | 0.051 | -0.022 | 0.000 | 0.000 | 0.028 | 0.000 | 0.029 | 0.000 |
| GHQ 5 | 0.040 | 0.035 | 0.000 | 0.000 | 0.124 | 0.054 | 0.000 | 0.000 | 0.059 | 0.026 | 0.000 | 0.000 | 0.000 | 0.000 | 0.000 |
| GHQ 6 | 0.000 | 0.046 | 0.047 | 0.000 | 0.078 | 0.119 | 0.049 | 0.035 | 0.058 | 0.052 | 0.069 | 0.040 | 0.000 | 0.041 | 0.000 |
| GHQ 7 | 0.034 | 0.000 | 0.029 | 0.000 | 0.000 | 0.021 | 0.093 | 0.000 | 0.000 | -0.034 | 0.000 | 0.000 | 0.000 | 0.000 | 0.000 |
| GHQ 8 | 0.000 | 0.000 | 0.000 | 0.045 | 0.000 | 0.000 | 0.000 | 0.076 | -0.023 | 0.000 | -0.030 | 0.000 | 0.000 | 0.000 | 0.000 |
| GHQ 9 | 0.000 | 0.049 | 0.000 | 0.000 | 0.086 | 0.070 | 0.039 | 0.000 | 0.169 | 0.100 | 0.074 | 0.036 | 0.000 | 0.000 | 0.000 |
| GHQ 10 | 0.033 | 0.066 | 0.000 | 0.034 | 0.060 | 0.084 | 0.000 | 0.000 | 0.080 | 0.189 | 0.058 | 0.000 | 0.000 | 0.000 | 0.000 |
| GHQ 11 | 0.063 | 0.087 | 0.109 | 0.077 | 0.085 | 0.128 | 0.062 | 0.085 | 0.125 | 0.140 | 0.314 | 0.116 | 0.068 | -0.038 | 0.000 |
| GHQ 12 | 0.000 | 0.000 | 0.000 | 0.000 | -0.038 | -0.040 | 0.000 | 0.037 | -0.035 | -0.035 | -0.038 | 0.058 | 0.000 | -0.032 | 0.000 |
| ECO 1 | 0.000 | 0.000 | 0.000 | 0.000 | -0.019 | 0.000 | 0.000 | 0.000 | 0.000 | 0.000 | 0.016 | 0.000 | 0.274 | -0.030 | -0.055 |
| ECO 2 | 0.000 | 0.025 | 0.000 | 0.000 | 0.000 | 0.000 | 0.000 | -0.021 | 0.000 | 0.000 | 0.000 | 0.000 | -0.055 | 0.203 | 0.052 |
| ECO 3 | 0.000 | 0.000 | 0.000 | 0.000 | 0.000 | 0.000 | 0.000 | 0.000 | 0.000 | -0.017 | -0.017 | 0.000 | -0.081 | 0.067 | 0.269 |

Note. The estimates are edge weights of variables measured in wave 4 predicting variables measured in wave 10. Cross-construct associations highlighted. Model fit: χ2(df = 63) = 31.05, p = 1.000, RMSEA = .000 CFI = 1.00, TLI = 1.00. n = 6,804 in the regularized regression step and n = 8,586 in the non-regularized regression step.


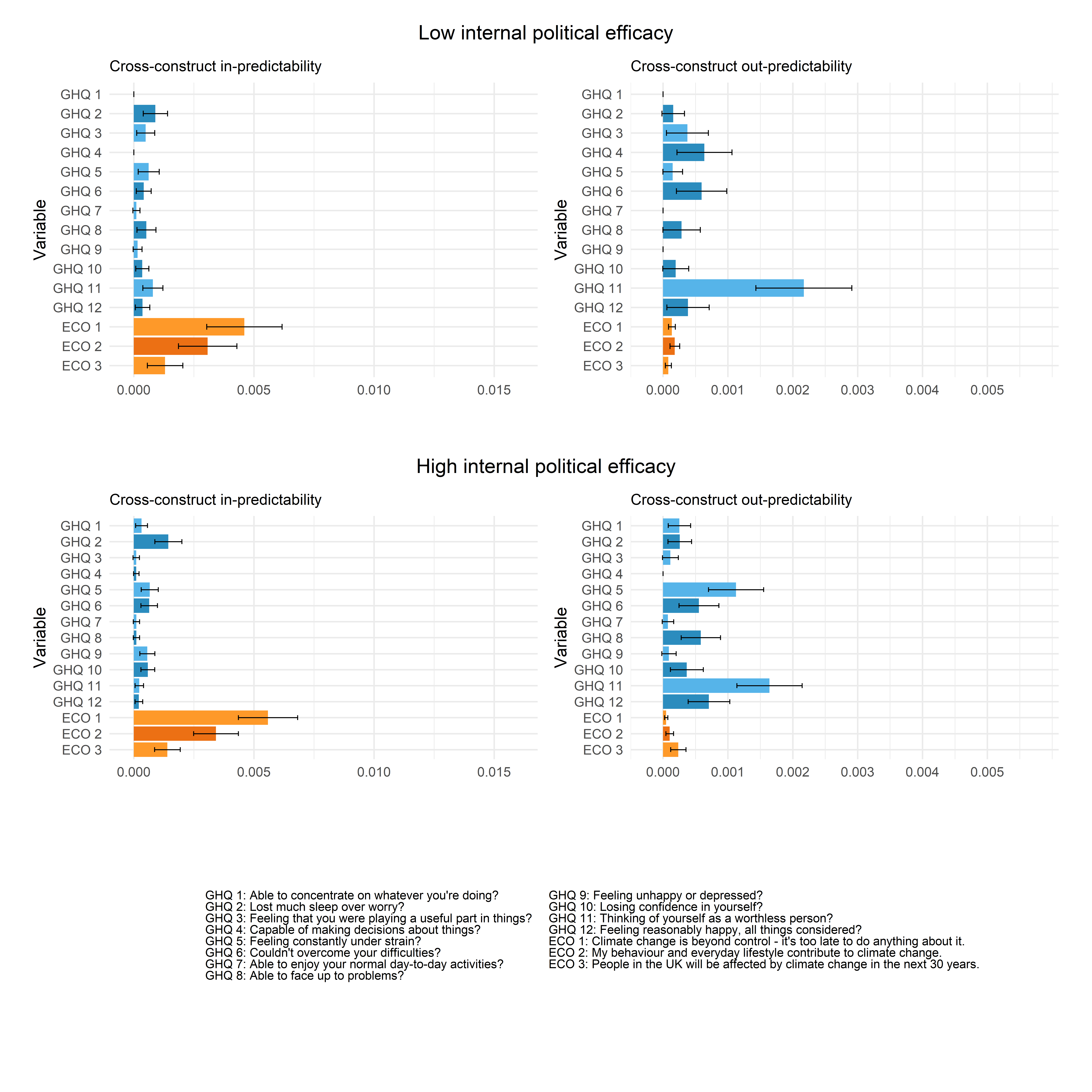
**S20** Cross-construct predictability metrics for each node in low vs. high internal political efficacy groups

**S21** The edge weights of the cross-lagged panel network for the high external political efficacy group

|  | Wave 10 | | | | | | | | | | | | | | |
| --- | --- | --- | --- | --- | --- | --- | --- | --- | --- | --- | --- | --- | --- | --- | --- |
| Wave 4 | GHQ 1 | GHQ 2 | GHQ 3 | GHQ 4 | GHQ 5 | GHQ 6 | GHQ 7 | GHQ 8 | GHQ 9 | GHQ 10 | GHQ 11 | GHQ 12 | ECO 1 | ECO 2 | ECO 3 |
| GHQ1 | 0.076 | 0.000 | 0.000 | 0.000 | 0.000 | 0.000 | 0.000 | 0.000 | 0.000 | 0.000 | 0.000 | 0.000 | 0.000 | 0.000 | 0.000 |
| GHQ 2 | 0.044 | 0.221 | 0.000 | 0.023 | 0.061 | 0.058 | 0.032 | 0.037 | 0.046 | 0.044 | 0.028 | 0.000 | 0.000 | 0.000 | 0.000 |
| GHQ 3 | 0.000 | 0.000 | 0.061 | 0.024 | 0.000 | 0.000 | 0.000 | 0.000 | 0.000 | 0.000 | 0.000 | 0.000 | 0.000 | 0.000 | 0.000 |
| GHQ 4 | 0.000 | -0.025 | 0.000 | 0.096 | 0.000 | 0.000 | 0.022 | 0.056 | 0.000 | 0.000 | 0.000 | 0.032 | 0.000 | 0.000 | 0.000 |
| GHQ 5 | 0.045 | 0.060 | 0.000 | 0.000 | 0.202 | 0.075 | 0.024 | 0.023 | 0.087 | 0.051 | 0.026 | 0.040 | -0.036 | 0.033 | 0.025 |
| GHQ 6 | 0.041 | 0.057 | 0.000 | 0.025 | 0.075 | 0.140 | 0.028 | 0.033 | 0.063 | 0.069 | 0.079 | 0.025 | 0.025 | 0.000 | 0.000 |
| GHQ 7 | 0.033 | 0.000 | 0.031 | 0.000 | 0.000 | 0.000 | 0.081 | 0.000 | 0.000 | -0.019 | 0.000 | 0.024 | 0.000 | 0.000 | 0.000 |
| GHQ 8 | 0.000 | 0.000 | 0.039 | 0.039 | -0.019 | 0.000 | 0.000 | 0.082 | -0.026 | -0.023 | -0.022 | 0.000 | -0.038 | 0.000 | -0.021 |
| GHQ 9 | 0.000 | 0.045 | 0.000 | 0.000 | 0.060 | 0.072 | 0.029 | 0.000 | 0.177 | 0.080 | 0.044 | 0.035 | 0.000 | 0.000 | 0.000 |
| GHQ 10 | 0.000 | 0.063 | 0.032 | 0.000 | 0.046 | 0.070 | 0.000 | 0.000 | 0.064 | 0.186 | 0.078 | -0.024 | 0.000 | 0.030 | 0.000 |
| GHQ 11 | 0.044 | 0.050 | 0.055 | 0.049 | 0.064 | 0.106 | 0.053 | 0.058 | 0.093 | 0.124 | 0.281 | 0.066 | 0.061 | 0.000 | 0.000 |
| GHQ 12 | 0.000 | -0.032 | 0.000 | 0.000 | -0.049 | -0.047 | 0.000 | 0.022 | -0.043 | -0.055 | -0.039 | 0.062 | 0.000 | -0.040 | 0.000 |
| ECO 1 | 0.018 | 0.000 | 0.000 | 0.000 | 0.000 | 0.022 | 0.000 | 0.023 | 0.000 | 0.000 | 0.026 | 0.000 | 0.310 | -0.047 | -0.053 |
| ECO 2 | 0.000 | 0.026 | 0.000 | 0.000 | 0.022 | 0.017 | 0.000 | 0.000 | 0.000 | 0.017 | 0.000 | 0.000 | -0.032 | 0.262 | 0.061 |
| ECO 3 | 0.000 | 0.022 | 0.000 | 0.000 | 0.000 | 0.014 | 0.000 | 0.000 | 0.020 | 0.000 | 0.000 | 0.016 | -0.060 | 0.075 | 0.302 |

Note. The estimates are edge weights of variables measured in wave 4 predicting variables measured in wave 10. Cross-construct associations highlighted. Model fit: χ2(df = 37) = 13.11, p = 1.000, RMSEA = .000 CFI = 1.00, TLI = 1.00. n = 12,564 in the regularized regression step and n = 14,843 in the non-regularized regression step.

**S22** The edge weights of the cross-lagged panel network for the low external political efficacy group

|  | Wave 10 | | | | | | | | | | | | | | |
| --- | --- | --- | --- | --- | --- | --- | --- | --- | --- | --- | --- | --- | --- | --- | --- |
| Wave 4 | GHQ 1 | GHQ 2 | GHQ 3 | GHQ 4 | GHQ 5 | GHQ 6 | GHQ 7 | GHQ 8 | GHQ 9 | GHQ 10 | GHQ 11 | GHQ 12 | ECO 1 | ECO 2 | ECO 3 |
| GHQ1 | 0.082 | 0.000 | 0.031 | 0.000 | 0.040 | 0.000 | 0.061 | 0.033 | 0.000 | 0.032 | 0.032 | 0.037 | 0.000 | 0.000 | 0.000 |
| GHQ 2 | 0.046 | 0.227 | 0.044 | 0.000 | 0.097 | 0.077 | 0.031 | 0.031 | 0.086 | 0.056 | 0.048 | 0.000 | 0.000 | 0.000 | 0.000 |
| GHQ 3 | 0.000 | 0.000 | 0.096 | 0.000 | 0.000 | 0.000 | 0.045 | 0.000 | 0.000 | 0.000 | 0.034 | 0.034 | 0.000 | -0.036 | 0.000 |
| GHQ 4 | 0.023 | -0.036 | 0.000 | 0.085 | -0.030 | 0.000 | 0.000 | 0.027 | -0.031 | 0.000 | -0.021 | 0.000 | 0.000 | 0.031 | 0.000 |
| GHQ 5 | 0.028 | 0.046 | 0.000 | 0.000 | 0.117 | 0.034 | 0.000 | 0.000 | 0.040 | 0.033 | 0.000 | 0.025 | 0.000 | 0.000 | 0.000 |
| GHQ 6 | 0.000 | 0.044 | 0.036 | 0.032 | 0.075 | 0.119 | 0.065 | 0.047 | 0.061 | 0.058 | 0.062 | 0.036 | 0.000 | 0.000 | 0.000 |
| GHQ 7 | 0.000 | 0.000 | 0.000 | 0.000 | 0.000 | 0.000 | 0.077 | 0.000 | 0.000 | -0.023 | 0.000 | 0.000 | -0.037 | 0.030 | 0.000 |
| GHQ 8 | 0.000 | 0.000 | 0.000 | 0.054 | 0.000 | 0.000 | 0.000 | 0.077 | -0.023 | 0.000 | 0.000 | 0.000 | 0.000 | -0.034 | 0.000 |
| GHQ 9 | 0.000 | 0.057 | 0.000 | 0.000 | 0.066 | 0.078 | 0.000 | 0.000 | 0.152 | 0.068 | 0.068 | 0.000 | 0.000 | 0.000 | 0.000 |
| GHQ 10 | 0.046 | 0.064 | 0.000 | 0.037 | 0.094 | 0.114 | 0.000 | 0.000 | 0.101 | 0.199 | 0.070 | 0.000 | 0.000 | 0.000 | 0.000 |
| GHQ 11 | 0.075 | 0.085 | 0.153 | 0.100 | 0.063 | 0.101 | 0.070 | 0.105 | 0.110 | 0.149 | 0.300 | 0.101 | 0.053 | 0.000 | 0.000 |
| GHQ 12 | 0.000 | 0.000 | 0.000 | 0.000 | -0.037 | -0.049 | 0.000 | 0.024 | -0.031 | -0.044 | -0.046 | 0.066 | 0.000 | 0.000 | 0.000 |
| ECO 1 | 0.000 | 0.000 | 0.000 | 0.000 | -0.022 | 0.000 | 0.000 | -0.020 | 0.000 | 0.000 | 0.000 | 0.000 | 0.252 | -0.038 | -0.040 |
| ECO 2 | 0.000 | 0.000 | 0.000 | 0.000 | 0.000 | 0.000 | 0.000 | 0.000 | 0.000 | 0.000 | 0.000 | 0.000 | -0.040 | 0.244 | 0.068 |
| ECO 3 | 0.000 | 0.000 | 0.000 | 0.000 | 0.000 | 0.000 | 0.000 | 0.000 | 0.000 | 0.000 | 0.000 | 0.000 | -0.050 | 0.087 | 0.290 |

Note. The estimates are edge weights of variables measured in wave 4 predicting variables measured in wave 10. Cross-construct associations highlighted. Model fit: χ2(df = 61) = 36.00, p = 0.996, RMSEA = .000 CFI = 1.00, TLI = 1.00. n = 7,530 in the regularized regression step and n = 9,355 in the non-regularized regression step.

**S23** Cross-construct predictability (in) and influence (out) for each node in low vs. high external political efficacy groups

**
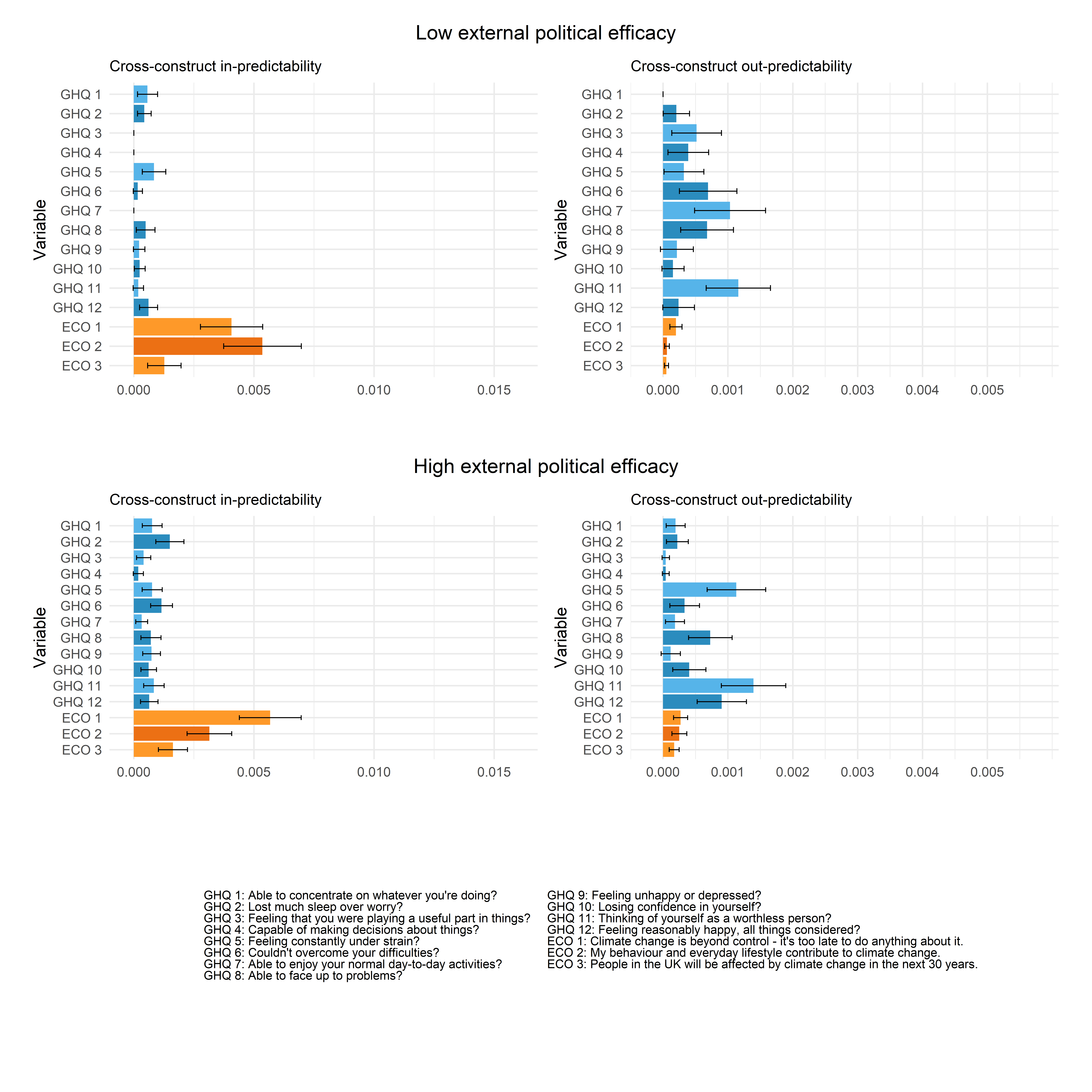
**
